# Interleukin 6 Increases Production of Cytokines by Colonic Innate Lymphoid Cells in Mice and Patients With Chronic Intestinal Inflammation

**DOI:** 10.1053/j.gastro.2015.04.017

**Published:** 2015-08

**Authors:** Nick Powell, Jonathan W. Lo, Paolo Biancheri, Anna Vossenkämper, Eirini Pantazi, Alan W. Walker, Emilie Stolarczyk, Francesca Ammoscato, Rimma Goldberg, Paul Scott, James B. Canavan, Esperanza Perucha, Natividad Garrido-Mesa, Peter M. Irving, Jeremy D. Sanderson, Bu Hayee, Jane K. Howard, Julian Parkhill, Thomas T. MacDonald, Graham M. Lord

**Affiliations:** 1 Department of Experimental Immunobiology, Division of Transplantation Immunology and Mucosal Biology, King’s College London, London, United Kingdom; 7 Division of Diabetes and Nutritional Sciences, King’s College London, London, United Kingdom; 2 Gastroenterology Department, Guy’s and St Thomas’ National Health Service Foundation Trust, London, United Kingdom; 3 National Institute for Health Research Biomedical Research Centre, Guy’s and St Thomas’ National Health Service Foundation Trust, London, United Kingdom; 4 Centre for Immunology and Infectious Disease, Blizard Institute, Barts and the London School of Medicine and Dentistry, London, United Kingdom; 5 Pathogen Genomics Group, Wellcome Trust Sanger Institute, Wellcome Trust Genome Campus, Cambridgeshire, United Kingdom; 6 Microbiology Group, Rowett Institute of Nutrition and Health, University of Aberdeen, Aberdeen, United Kingdom; 8 Gastroenterology Department, Kings College Hospital, London, United Kingdom

**Keywords:** UC, CD, Innate Immunity, Immune Regulation, CD, Crohn’s disease, cLPMC, colonic lamina propria mononuclear cell, ELISA, enzyme-linked immunosorbent assay, IBD, inflammatory bowel disease, IL, interleukin, ILC, innate lymphoid cell, IL7R^+^, IL7R-receptor–positive, mLN, mesenteric lymph node, NCR, natural cytotoxicity receptor, OTU, operational taxonomic unit, PCR, polymerase chain reaction, PMA, phorbol 12-myristate 13-acetate, sIL6Rα, soluble interleukin 6Rα, Th, T-helper cell, TRUC, *Tbx21*^*-/-*^*Rag2*^*-/-*^ ulcerative colitis, UC, ulcerative colitis

## Abstract

**Background & Aims:**

Innate lymphoid cells (ILCs) are a heterogeneous group of mucosal inflammatory cells that participate in chronic intestinal inflammation. We investigated the role of interleukin 6 (IL6) in inducing activation of ILCs in mice and in human beings with chronic intestinal inflammation.

**Methods:**

ILCs were isolated from colons of *Tbx21*^-/-^ × *Rag2*^-/-^ mice (TRUC), which develop colitis; patients with inflammatory bowel disease (IBD); and patients without colon inflammation (controls). ILCs were characterized by flow cytometry; cytokine production was measured by enzyme-linked immunosorbent assay and cytokine bead arrays. Mice were given intraperitoneal injections of depleting (CD4, CD90), neutralizing (IL6), or control antibodies. Isolated colon tissues were analyzed by histology, explant organ culture, and cell culture. Bacterial DNA was extracted from mouse fecal samples to assess the intestinal microbiota.

**Results:**

IL17A- and IL22-producing, natural cytotoxicity receptor–negative, ILC3 were the major subset of ILCs detected in colons of TRUC mice. Combinations of IL23 and IL1α induced production of cytokines by these cells, which increased further after administration of IL6. Antibodies against IL6 reduced colitis in TRUC mice without significantly affecting the structure of their intestinal microbiota. Addition of IL6 increased production of IL17A, IL22, and interferon-γ by human intestinal CD3-negative, IL7-receptor–positive cells, in a dose-dependent manner.

**Conclusions:**

IL6 contributes to activation of colonic natural cytotoxicity receptor–negative, CD4-negative, ILC3s in mice with chronic intestinal inflammation (TRUC mice) by increasing IL23- and IL1α-induced production of IL17A and IL22. This pathway might be targeted to treat patients with IBD because IL6, which is highly produced in colonic tissue by some IBD patients, also increased the production of IL17A, IL22, and interferon-γ by cultured human colon CD3-negative, IL7-receptor–positive cells.

Inflammatory bowel disease (IBD), comprising Crohn’s disease (CD) and ulcerative colitis (UC), is an increasingly common immune-mediated disease of the gut of unknown cause.^1^, ^2^ The genetic architecture of IBD is complex, with more than 130 significantly associated susceptibility loci identified to date,^3^ indicating that multiple mechanisms of disease may exist. Nevertheless, prominent roles for innate immunity and particular immune response pathways, including the interleukin (IL) 23/IL17 axis, strongly are implicated.

Innate lymphoid cells (ILCs) are emerging as important players in mucosal immunity. Although recognized to perform protective roles against mucosal pathogens,^4^, ^5^ they also contribute to chronic intestinal inflammation, which is particularly apparent in mice lacking conventional T and B cells.^6^, ^7^ This is in part dependent on their capacity to produce inflammatory cytokines, including interferon-γ, IL17A, and IL22.^4^, ^5^, ^6^, ^7^, ^8^ ILCs can be subdivided into discrete populations, which accumulate in mucosal tissues in different pathologic settings.^9^ At least 3 subsets exist, including ILC1s, which produce interferon-γ; ILC2s, which produce IL5/IL13; and ILC3, which can be subdivided further based on differential expression of natural cytotoxicity receptors (NCRs), CD4, and production of IL17 and/or IL22.^9^

*Tbx21*^*-/-*^*Rag2*^*-/-*^ ulcerative colitis (TRUC) mice spontaneously develop severe colitis with striking similarities to some aspects of human UC.^10^ Colon lesions histologically resemble UC with goblet cell depletion, crypt abscess formation, epithelial hyperplasia, and infiltration of colonic lamina propria with neutrophils and mononuclear cells.^7^, ^10^ TRUC mice develop inflammation-associated epithelial dysplasia, which frequently progresses to frank adenocarcinoma,^11^ one of the most severe complications in human forms of IBD. TRUC disease is dependent on interactions between intestinal CD11c^+^ mononuclear phagocytes and CD90^+^ IL7R-receptor–positive (IL7R^+^) ILCs.^7^ Depletion of ILCs or genetic deficiency of the common γ-chain cytokine receptor, which is necessary for ILC survival, prevents disease.^7^ Similarly, blockade of IL23 or IL17A significantly attenuates disease.^7^ ILCs accumulate in gut lesions from IBD patients^12^, ^13^, ^14^ and it has been speculated that targeting these cells might represent a viable therapeutic approach in IBD.^15^ IL23^6^, ^7^ and IL1β^16^ contribute to ILC activation, although curiously TRUC mice that additionally are deficient for either IL23R or IL1R are not fully protected from colitis,^17^ consistent with a possible role for alternative ILC activation pathways contributing to disease. The purpose of this study was to investigate the proximal signals responsible for driving intestinal ILC activation and to determine whether similar pathways might exist in human disease.

## Materials and Methods

### Mice

Balb/C *Rag2*^-/-^ and wild-type mice were sourced commercially (Jackson Laboratories; Bar Harbor, ME). TRUC mice were a gift from Laurie Glimcher. Animal experiments were performed in accredited facilities in accordance with the UK Animals (Scientific Procedures) Act 1986 (Home Office License Number PPL: 70/6792 and PPL: 70/7869 from November 2013).

### Human Studies

Studies in human tissues received ethical approval from the City and Hackney Local Research Ethics Committee (REC reference: 10/H0704/74 and 10/H0804/65). Colonic lamina propria mononuclear cells (cLPMCs) were isolated as described previously^18^ from colectomy specimens and endoscopically acquired biopsy specimens. Normal colonic mucosal samples were collected from macroscopically unaffected areas of patients undergoing intestinal resection for colon cancer or polyps. Informed written consent was obtained in all cases.

### Flow Cytometry and Cell Sorting

Intracellular cytokine expression was measured as described previously.^7^ Cells were stimulated with IL23 (10–20 ng/mL), IL6 (10–100 ng/mL), or phorbol 12-myristate 13-acetate (PMA) (50 ng/mL) and ionomycin (1 μmol/L) for 4–6 hours at 37°C with monensin (3 μmol/L) added for the last 2 hours. In human work, antibodies used to stain cell surface antigens were incubated with unstimulated cells for 25 minutes and then fixed in 2% paraformaldehyde pending analysis. For fluorescence-activated cell sorter purification of murine ILCs, CD45^+^ cells first were sorted immunomagnetically from unfractionated cLPMCs using anti-CD45 beads (Miltenyi) and LS columns (Miltenyi). CD45^+^ cells were stained with CD90, NKp46, and IL7R. Antibodies used in flow cytometry experiments are listed in Supplementary Table 1.

### Ex Vivo Organ Culture

Colon explants cultures from murine experiments were performed as described previously.^7^ Three biopsy punches from the distal colon were cultured in 500 μL of complete medium for 24 hours at 37°C. In human studies explant cultures were set up as described previously.^18^ Cytokine production in culture supernatants was measured by enzyme-linked immunosorbent assay (ELISA).

### Cell Culture

Unfractionated murine splenocytes (2 × 10^6^/mL) and mesenteric lymph node (mLN) cells (1 × 10^6^/mL) or cLPMCs (1 × 10^6^/mL) were cultured in complete medium for 24 hours at 37°C as described previously.^7^ cLPMCs from IBD and noninflammatory control patients were cultured with recombinant human IL6 (R&D) (0–100 ng/mL) overnight at 37°C, 5% CO_2_, and then restimulated with PMA (50 ng/mL) and ionomycin (1 μmol/L). In some experiments, cLPMCs were cultured with IL6 (100 ng/mL) for 6 hours in the presence of monensin (3 μmol/L). Fluorescence-activated cell sorter–purified NCR^-^ ILC3s (CD45^+^ CD90^+^ IL7R^+^ NKp46^-^) from TRUC mice were cultured at 5 × 10^4^/mL for 24 hours. Cytokine concentrations in culture supernatants were measured by ELISA (R&D Systems and eBioscience).

### Histology

Colon histology was processed, stained (H&E), and colitis scores were calculated as described previously.^7^ Proximal and distal colitis scores from individual mice were averaged, unless otherwise stated.

### ELISA and Cytokine Bead Arrays

Cytokine concentrations were measured in culture supernatants by ELISA or T-helper cell (Th)1, Th2, or Th17 CBA (BD Biosciences).

### Microarray and Real-Time Polymerase Chain Reaction

RNA was extracted from 3 *Rag2*^-/-^ and 3 TRUC mice, aged 10 weeks, using TRIzol reagent (Invitrogen Carlsbad, CA). Transcript expression was analyzed with Mouse Genome 430 2.0 Affymetrix Expression Array. For real-time polymerase chain reaction (PCR) experiments cells were lysed in TRIzol reagent (Invitrogen) and RNA was extracted. Complementary DNA was generated with the complementary DNA synthesis kit (Bioline, Taunton, MA). Quantitative PCR was used to quantify messenger RNA transcripts using TaqMan gene expression assays (Applied Biosystems). Gene expression was normalized to the expression of β-actin to generate ΔCT values and relative abundance was quantified using the 2^-ΔCT^ method. Human RORC (Hs01076112_m1) and β-actin (4326315E) TaqMan quantitative PCR primer sets were used.

### In Vivo Antibody Treatment

Intraperitoneal injections of anti-CD4 (1 mg, GK1.5), anti-CD90 (1 mg, 30H12), anti-IL6 (750 μg, MP5-20F3), or isotype-matched control antibodies (LTF-2 or HRPN) (Bio X Cell, West Lebanon, NH) were administered on days 0, 7, 14, 21, and 28 (anti-CD4, anti-CD90) or days 0, 4, 9, 14, 18, 23, and 27 (anti-IL6).

### Microbiota Analysis

See the Supplementary Materials and Methods section for more detail.

## Results

### NCR^-^ CD4^-^ ILC3 Cells Are the Predominant Colonic ILC Subset in Chronic Intestinal Inflammation in TRUC Mice

**Figure 1:**
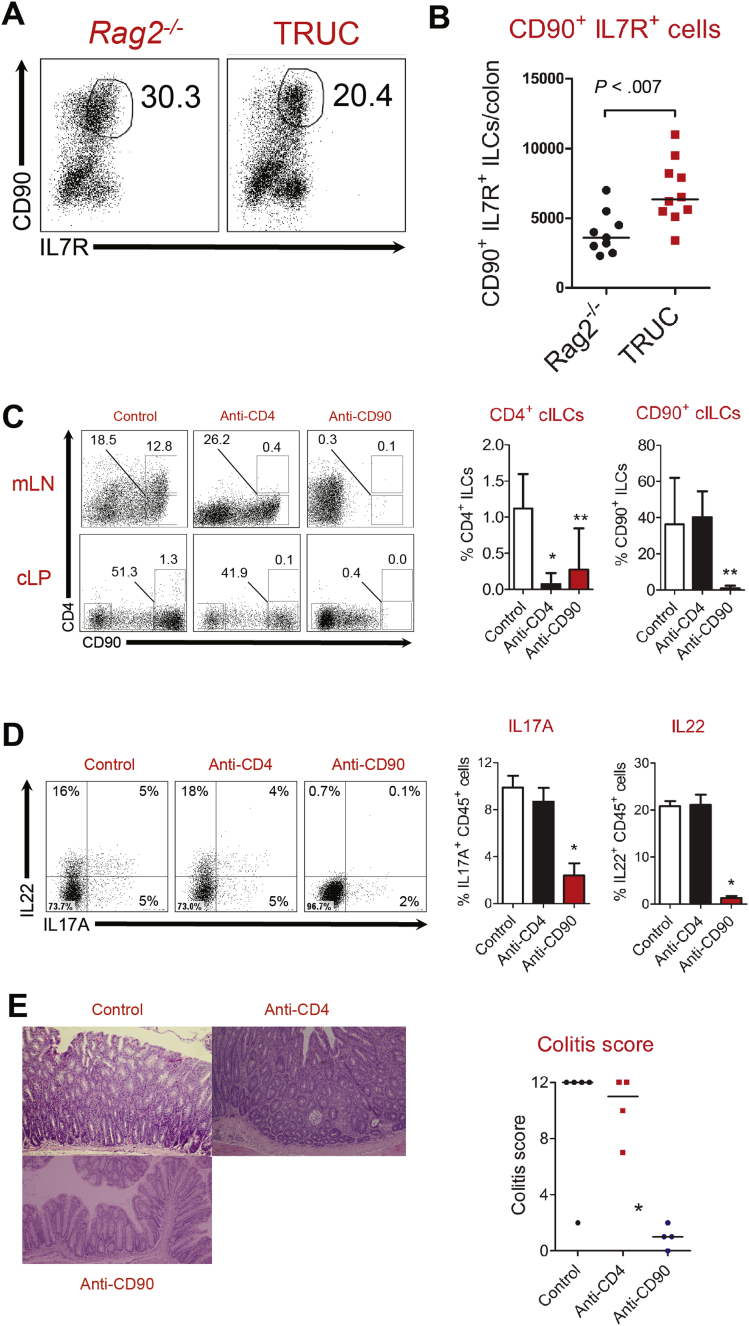
IL17A/IL22-producing CD4^-^ NCR^-^ ILC3 mediate colitis in TRUC mice. (*A*) Flow cytometry dot plots of live, CD45^+^ cells according to expression of CD90 and IL7R in the colons of *Rag2*^-/-^ and TRUC mice, and (*B*) absolute numbers of live CD45^+^ CD90^+^ IL7R^+^ ILCs in the colons of TRUC and *Rag2*^-/-^ mice. Each *dot/square* represents an individual mouse. *Line* depicts the median. (*C*) Representative flow cytometry dot plots (*left*) and statistical analysis (*right*) of the proportion of CD4^+^ and CD90^+^ cells (gated on live CD45^+^ cells) in mLNs and colons of TRUC mice treated with isotype-matched control antibodies (n = 5), anti-CD4 (n = 4), or anti-CD90 (n = 4) antibodies. Statistical analyses were performed on colonic cells and show the proportion of colonic ILCs (cILCs) after treatment. *P < .019, **P < .04. (*D*) Representative flow cytometry dot plots (*left*) and statistical analysis (*right*) of the proportion of IL17A^+^ and IL22^+^ cells (gated on live CD45^+^ cells) in the colon of TRUC mice treated with isotype-matched control antibodies (n = 5), anti-CD4 (n = 4), or anti-CD90 (n = 24) antibodies. Cells were stimulated with PMA and ionomycin before intracellular cytokine staining *P < .01. (*E*) Representative colon micrographs (H&E stained) (*left*) and statistical analysis of colitis scores (*right*) of TRUC mice treated with isotype-matched control antibodies, anti-CD4, or anti-CD90 antibodies. *P < .03 (for both anti-CD90 vs control antibody and anti-CD90 vs anti-CD4). Each *dot/square* represents an individual mouse. *Lines* depict the median.

**Supplementary Figure 1:**
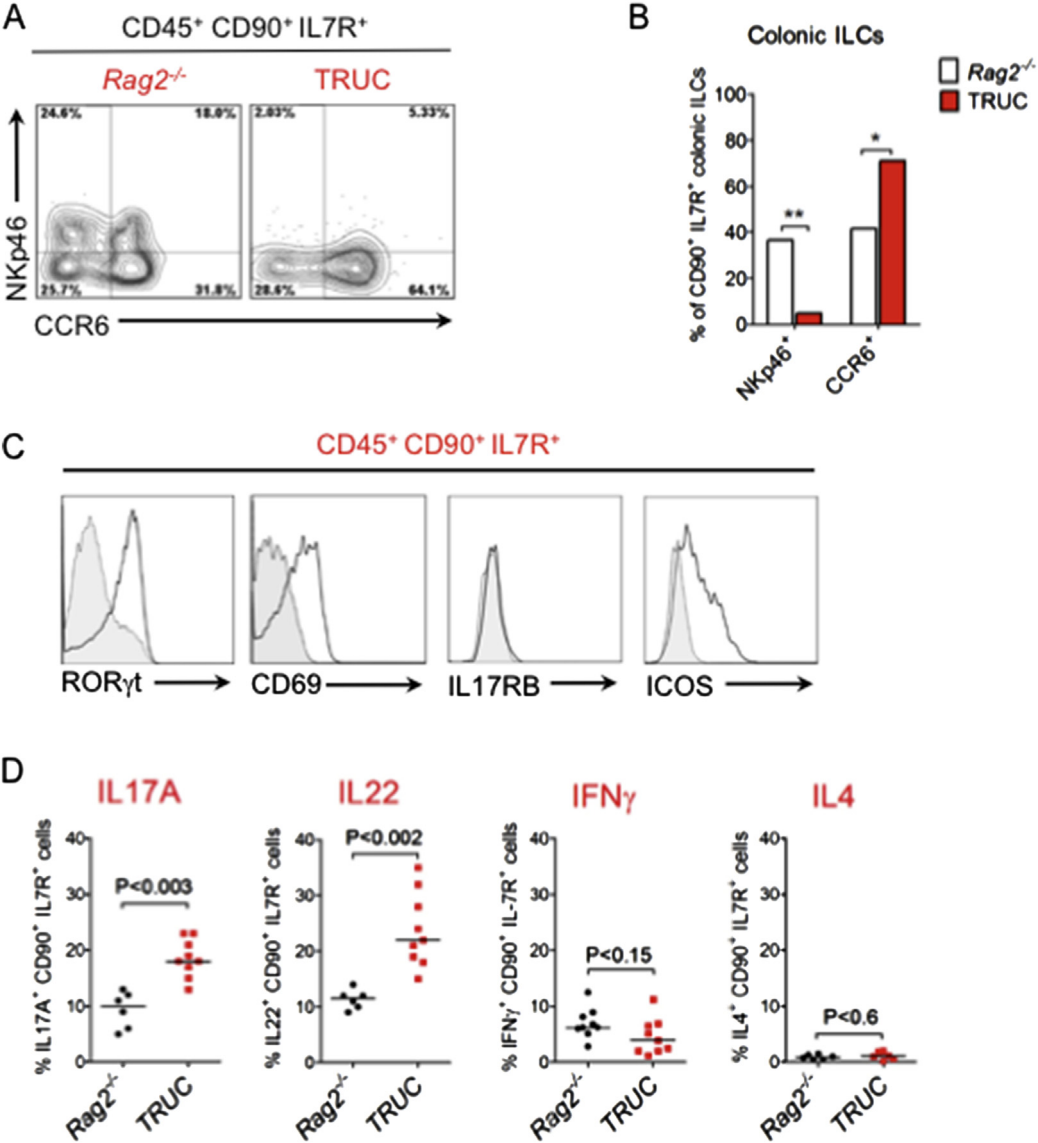
(*A*) Representative flow cytometry plot and statistical analysis (*B*) showing differential expression of NKp46 and CCR6 in live CD45^+^ CD90^+^ IL7R^+^ cells in the colon of *Rag2*^-/-^ (n = 6, *white bar*) and TRUC (n = 5, *red bar*) mice. *Error bars* depict SEM. *P < .01, **P < .005. (*C*) Flow cytometric analysis of the phenotype of CD90^+^ IL7R^+^ ILCs in the colons of TRUC mice. *Grey histograms* show staining with isotype-matched control antibody in comparison with staining with specific antibody (*white histograms with black lines*). Data are representative of more than 3 individual experiments. (*D*) Proportion of cytokine-expressing CD45^+^ CD90^+^ IL7R^+^ ILCs in the colons of *Rag2*^-/-^ and TRUC mice after stimulation of unfractionated cLPMCs with PMA and ionomycin. Each *dot/square* represents an individual mouse.

We validated the phenotype of ILCs in TRUC mice, confirming excessive accumulation of IL17A- and IL22-producing CD90^+^ IL7R^+^ NCR^-^ ILC3 in diseased colons (Figure 1*A* and 1*B*, Supplementary Figure 1*A–D*). CD4-expressing NCR^-^ ILC3s resembling lymphoid tissue inducer cells participate in mucosal immune responses in the gut,^5^ therefore, we considered the possibility that CD4^+^ ILCs might be the NCR^-^ ILC3 subset responsible for mediating chronic inflammation in TRUC mice. CD4^+^ cells were present in mLNs of TRUC mice (many of which co-expressed CD90); however, very few CD4^+^ cells were present in the colon (Figure 1*C*). Given the low frequency of intestinal CD4^+^ ILCs in TRUC mice we considered it unlikely that these cells would play a major role in disease. To test this assumption we depleted CD4-expressing cells in vivo. The administration of anti-CD4 antibodies successfully depleted CD4-expressing cells in mLNs and colon of TRUC mice (Figure 1*C*). However, many CD90^+^ cells still remained in the colon and there was no reduction in the number of IL17A- or IL22-producing cells (Figure 1*D*). Depleting anti-CD4 treatment did not alter the severity of TRUC disease significantly (Figure 1*E*). In contrast, anti-CD90 treatment depleted both CD90- and CD4-expressing ILCs, reduced the number of IL17- and IL22-producing cells in the colon, and significantly attenuated disease (Figure 1*C–E*). Taken together, these data indicate IL17A/IL22 producing CD90^+^ IL7R^+^ NCR^-^ CD4^-^ ILC3 are the key ILC population in the colon responsible for causing disease in TRUC mice.

### IL6 Is Expressed Highly and Augments Pathogenic Cytokine Production in TRUC Mice

**Figure 2:**
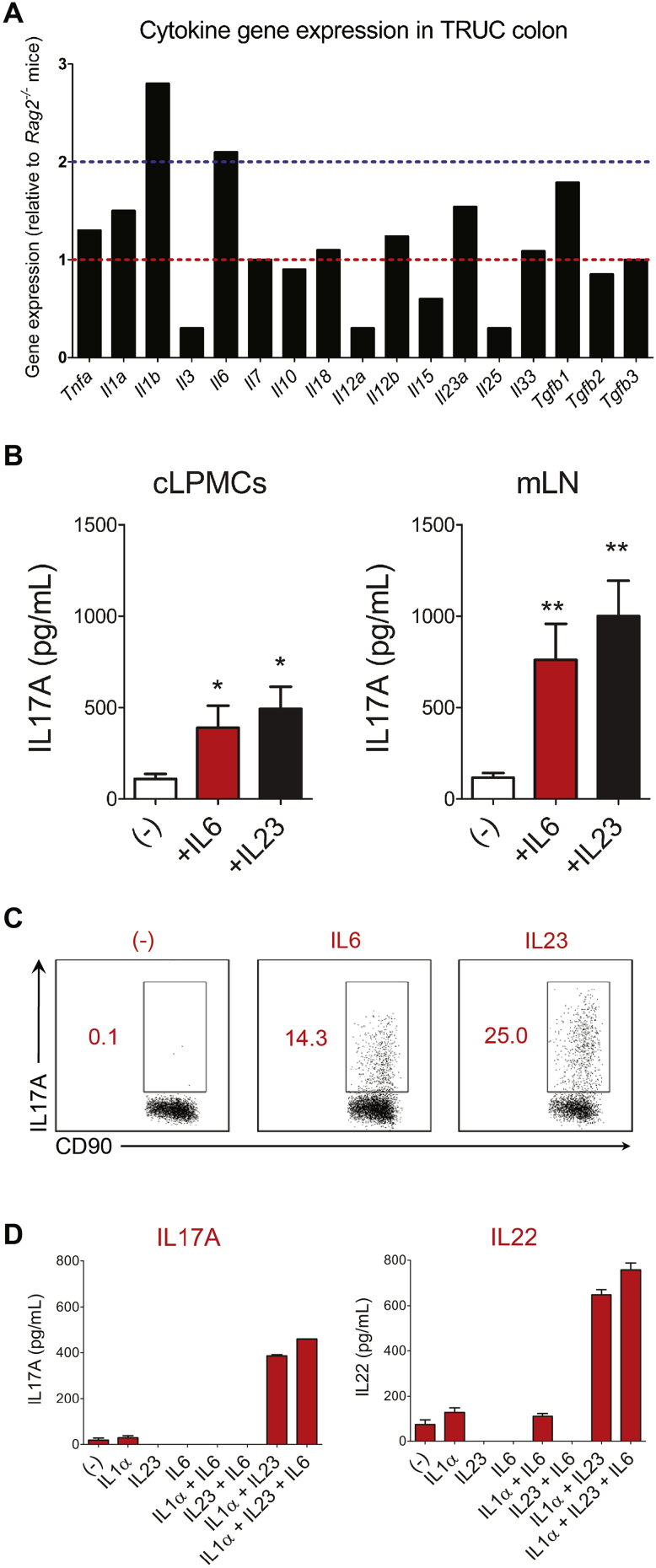
IL6 promotes cytokine production by NCR^-^ ILC3s in a cell-intrinsic manner. (*A*) Microarray analysis showing an abundance of cytokine transcripts in the colon of TRUC mice relative to *Rag2*^-/-^ mice. *Blue dotted line* depicts 2-fold induction. (*B*) IL17A production by unfractionated cLPMCs and mLN cells isolated from TRUC mice in medium alone (-) or after supplementation with recombinant IL6 or IL23. *Columns* represent mean cytokines and *error bars* depict SEM. Analysis of cLPMCs comprised 4 biological replicates. Analysis of mLN included 9 biological replicates for the unstimulated condition and 7 biological replicates for each of the stimulated conditions. *P < .02; **P < .003. (*C*) Flow cytometry plots of intracellular IL17A expression by CD90^+^ NKp46^-^ cells after stimulation of unfractionated mLN cells with IL6, IL23, or unstimulated cells (-), which were incubated with monensin alone. Data are representative of 3 separate experiments. (*D*) Cytokine production by fluorescence-activated cell sorted CD45^+^ CD90^+^ IL7R^+^ NKp46^-^ ILCs purified from the colons of TRUC mice (the gating strategy for cell sorting is illustrated in Supplementary Figure 3*A*). Purified NCR^-^ ILCs were stimulated with combinations of IL1α, IL6, and IL23 as depicted. After 24 hours cytokine concentrations were measured in culture supernatant by ELISA or CBA. Data are representative of 2 individual experiments with ILCs pooled from 10–15 colons. *Bars* show the mean cytokine production and *error bars* depict SEM.

We sought to define the proximal immune signals responsible for triggering effector function of colonic NCR^-^ ILC3 in TRUC mice. IL1β and IL6 were among the most highly expressed (>2-fold induction) cytokine transcripts in the colon of TRUC mice in comparison with *Rag2*^*-/-*^ mice (Figure 2*A*). The other IL1 family member, *Il1a*, and the IL23 subunit transcripts (*Il23a* and *Il12b*) also were increased. Proximal cytokines responsible for driving ILC1 (IL12, IL15, and IL18) or ILC2 (IL25 and IL33) responses were not up-regulated, and, indeed in most instances were down-regulated in the colon of TRUC mice in comparison with *Rag2*^*-/-*^ controls.

**Supplementary Figure 2:**
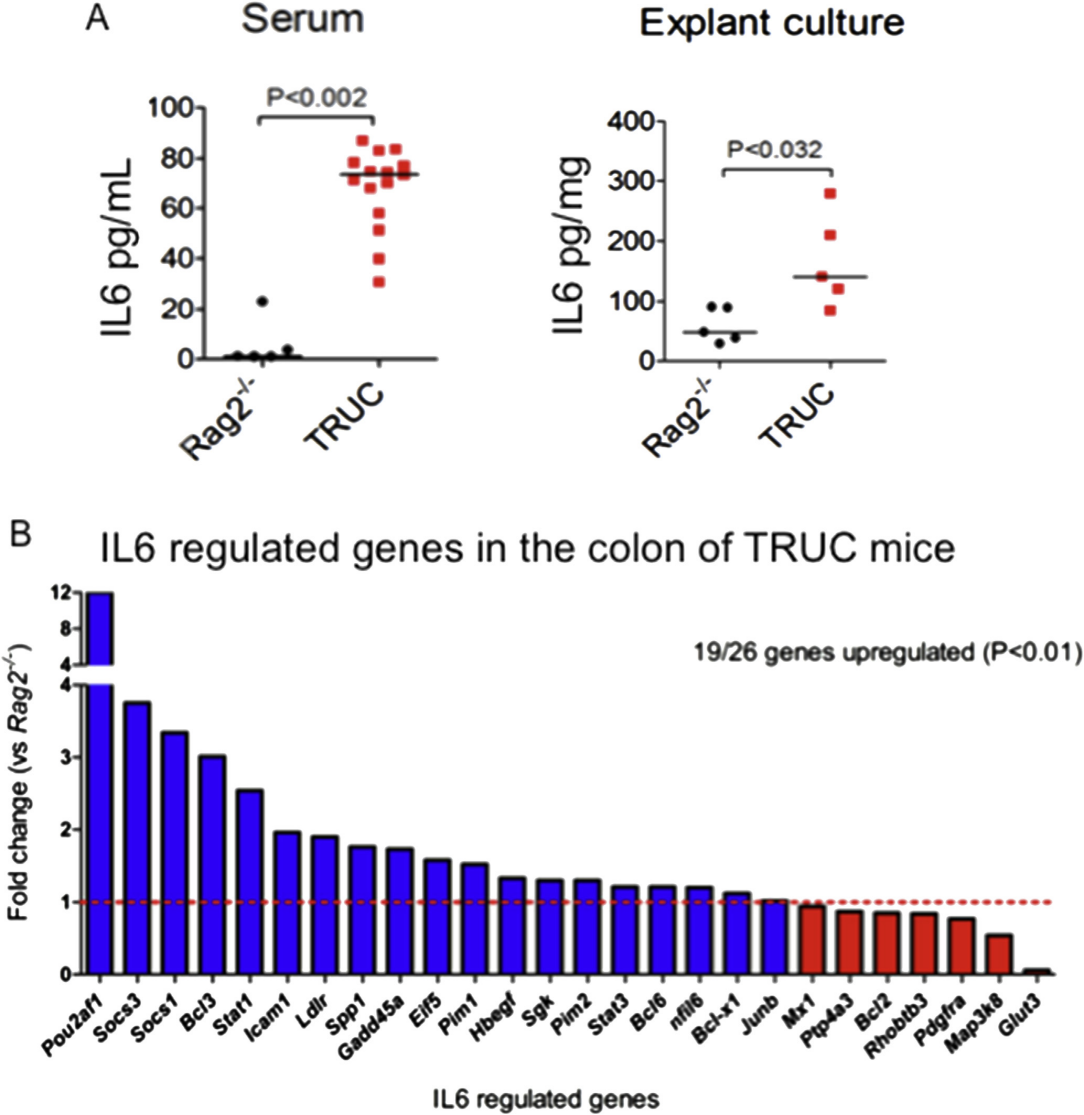
(*A*) IL6 concentration in serum (*left panel*) and colon explant culture (*right panel*) of *Rag2*^-/-^ and TRUC mice measured by ELISA. *Dots/squares* represent individual mice. *Line* depicts the median. (*B*) Microarray analysis showing abundance of transcripts encoded by genes known to be regulated by IL6 in the colon of TRUC mice relative to *Rag2*^-/-^ mice. *Blue bars* represent transcripts up-regulated in the colons of TRUC mice and *red bars* represent down-regulated genes (in comparison with *Rag2*^-/-^ mice).

IL23 and IL1 have been described to play an important role triggering ILCs in TRUC disease, therefore, in this study we focussed our attention on IL6. In addition to increased *Il6* transcripts in the colon, there were very high concentrations of IL6 in serum and significantly increased production of IL6 in colon explant cultures from TRUC mice (Supplementary Figure 2*A*). Transcripts of genes known to be regulated by IL6^19^ were up-regulated in the colon of TRUC mice in comparison with *Rag2*^*-/-*^ controls (Supplementary Figure 2*B*), including well-recognized immune genes (*Socs1*, *Socs3*, and *Icam1*), IL6 signaling components (*Stat1* and *Stat3*), and anti-apoptotic genes (*Bcl3*, *Bcl6*, and *Bcl-x1*). The most highly expressed IL6-regulated gene in the colon of TRUC mice (12-fold enrichment) was *Pou2af1*, which encodes a transcriptional co-activator responsible for IL6-mediated regulation of IL17 responses in T cells.^20^

**Supplementary Figure 3:**
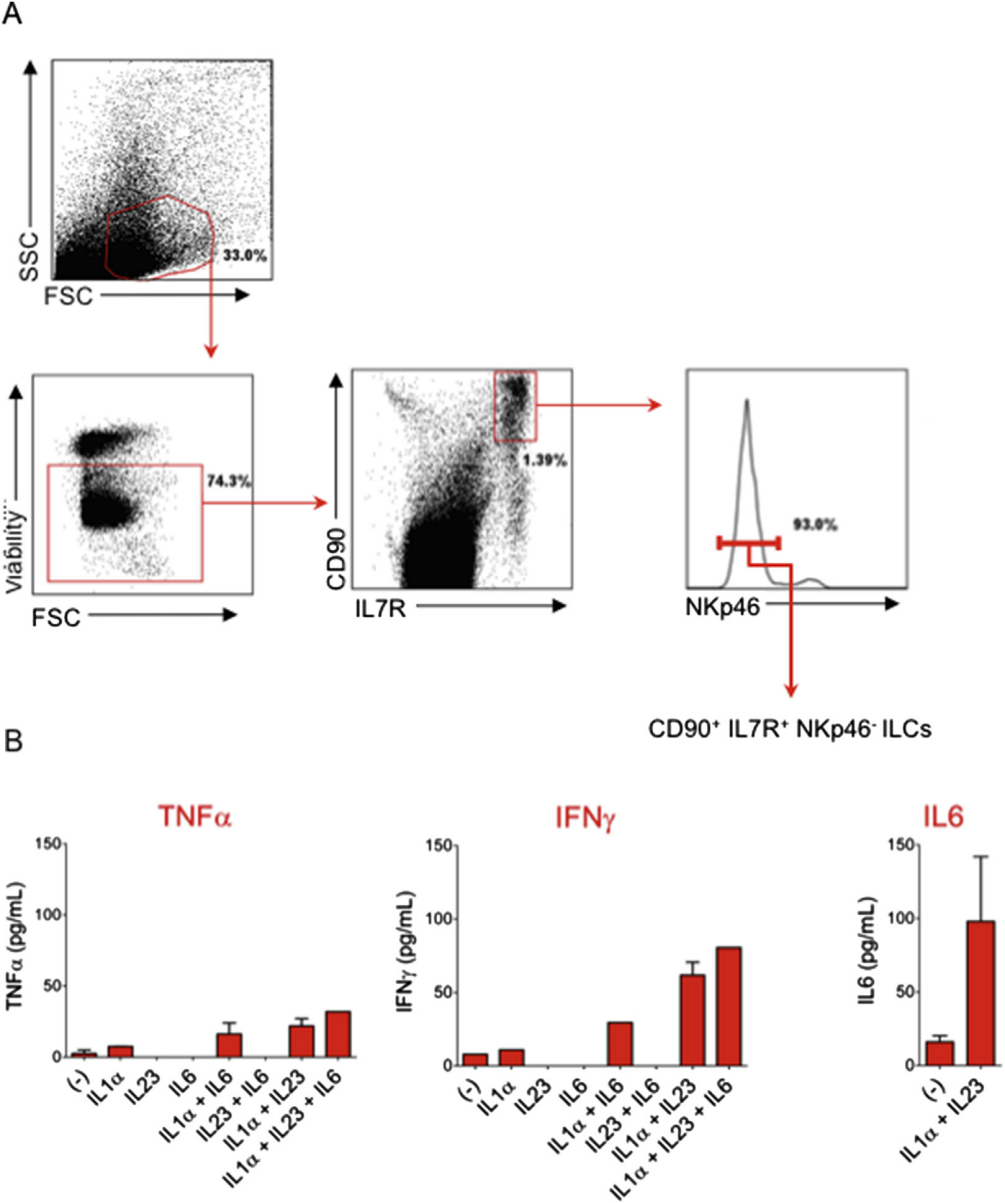
(*A*) FACs gating strategy used to purify ILCs from the colon of TRUC mice. CD45^+^ cells were first enriched from cLPMCs using anti-CD45 immunomagnetic beads. ILCs were FACs purified more than 98%. (*B*) Cytokine production by FACs purified CD90^+^ IL7R^+^ NKp46^-^ colonic ILCs from TRUC mice. Cells were cultured in the presence of combinations of IL1α, IL23, IL6 (as depicted), or medium alone (unstimulated). IL6 was measured in culture supernatant by CBA. *Bars* show the mean cytokine production and *error bars* depict the SEM. Data are from 2 independent experiments.

To determine whether IL6 might trigger ILC-derived cytokines we stimulated unfractionated cLPMCs and mLN cells from TRUC mice with recombinant IL6. Strikingly, IL6 triggered IL17A production by both cLPMCs and mLN cells (Figure 2*B*). We also performed flow cytometry with intracellular cytokine staining after IL6 stimulation of unfractionated mLN cells. Although less potent than IL23, IL6 induced expression of IL17A in NCR^-^ ILC3s (Figure 2*C*). To determine whether this was a cell-intrinsic phenomenon we purified CD90^+^ IL7R^+^ NCR^-^ ILC3s from TRUC colons by fluorescence-activated cell sorting (Supplementary Figure 3*A*). To our surprise, neither IL6, IL23, nor IL1α by themselves induced significant cytokine production by purified colonic NCR^-^ ILC3s (Figure 2*D*). However, the combination of IL23 and IL1α was a potent trigger for ILC production of IL17A and IL22. The addition of IL6 together with IL23 and IL1α was the most potent trigger of all. Purified intestinal NCR^-^ ILCs from TRUC mice produced little tumor necrosis factor α or interferon-γ under these conditions (Supplementary Figure 3*B*). IL23 and IL1α were weak inducers of IL6 by colonic NCR^-^ ILCs (Supplementary Figure 3*B*). Taken together, these data showed that IL6 augments IL23/IL1α-induced pathogenic cytokine production by intestinal ILCs in TRUC mice in a cell-intrinsic manner.

**Supplementary Figure 4:**
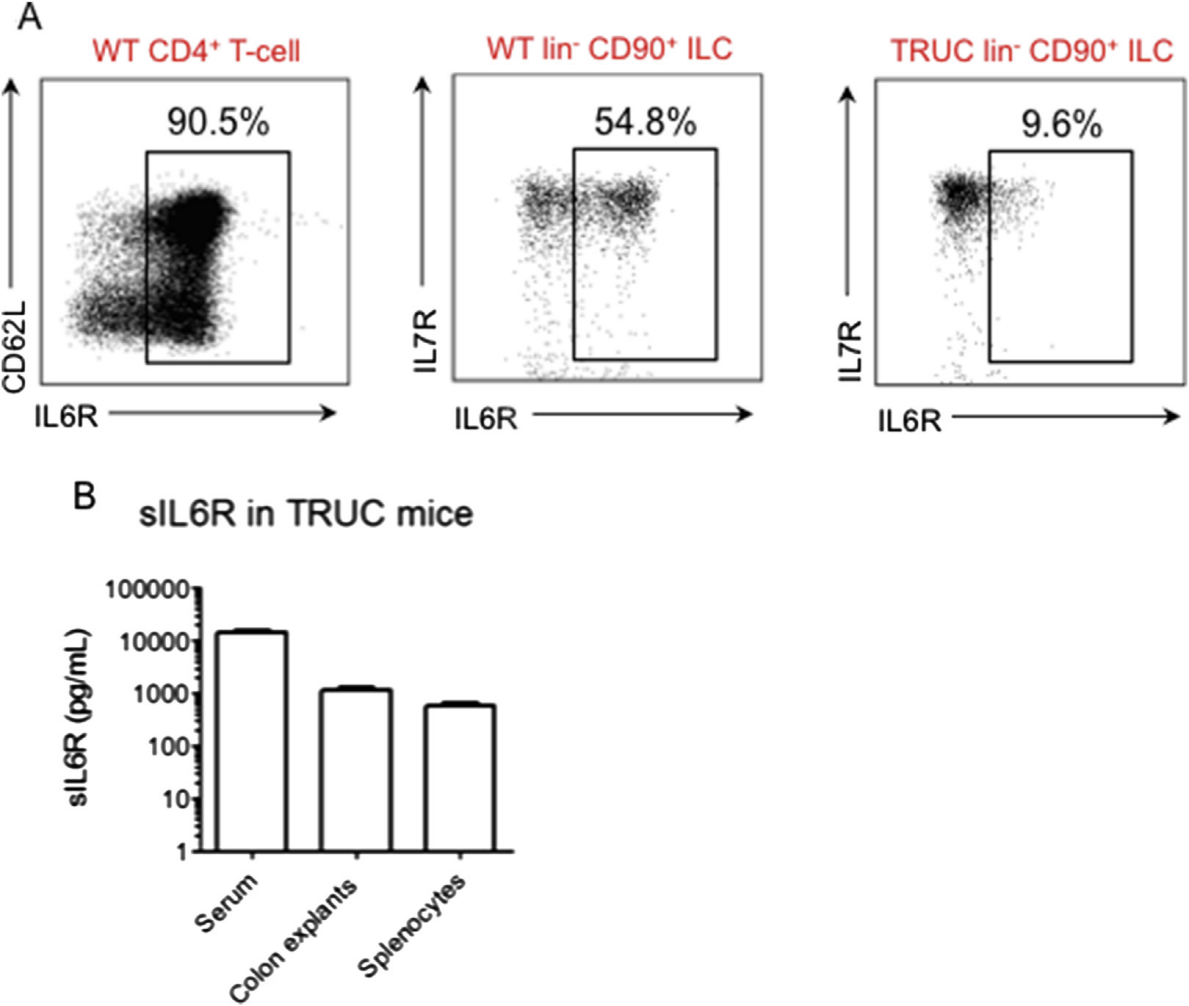
(*A*) Flow cytometry plots showing IL6R expression by CD4^+^ T cells, ILCs (lineage^-^ IL7R^+^) in the colons of wild-type (WT) and TRUC mice. Data are representative of 3 independent experiments. (*B*) Concentration of sIL6R in serum (n = 6) and supernatants of cultured colon explants (n = 5) and unfractionated splenocytes (n = 3) measured by ELISA. *Bars* represent the mean sIL6R concentration and *error bars* depict the SEM.

IL6 signals through a heterodimeric receptor comprising ubiquitously expressed gp130 and selectively expressed IL6Rα. However, IL6Rα also exists as a soluble form, which can complex with IL6 in solution and then bind to cells expressing gp130, enabling cells, which do not usually express IL6Rα, to respond to IL6 stimulation. Therefore, we investigated IL6Rα and soluble IL6Rα (sIL6Rα) expression in TRUC mice. IL6Rα expression by ILCs was highly variable in the colon of TRUC mice, but typically was less than 10% (Supplementary Figure 4*A*, and data not shown). However, sIL6Rα was abundant in the serum of TRUC mice and was detected in supernatants from cultured colon explants and unfractionated splenocytes (Supplementary Figure 4*B*). Therefore, it is likely that ILCs respond to IL6 stimulation directly, but also potentially through trans-signaling given the abundance of sIL6R in TRUC mice.

### IL6 Blockade Attenuated TRUC Disease Independently of Changes to Intestinal Microbiota Community Profiles

**Figure 3:**
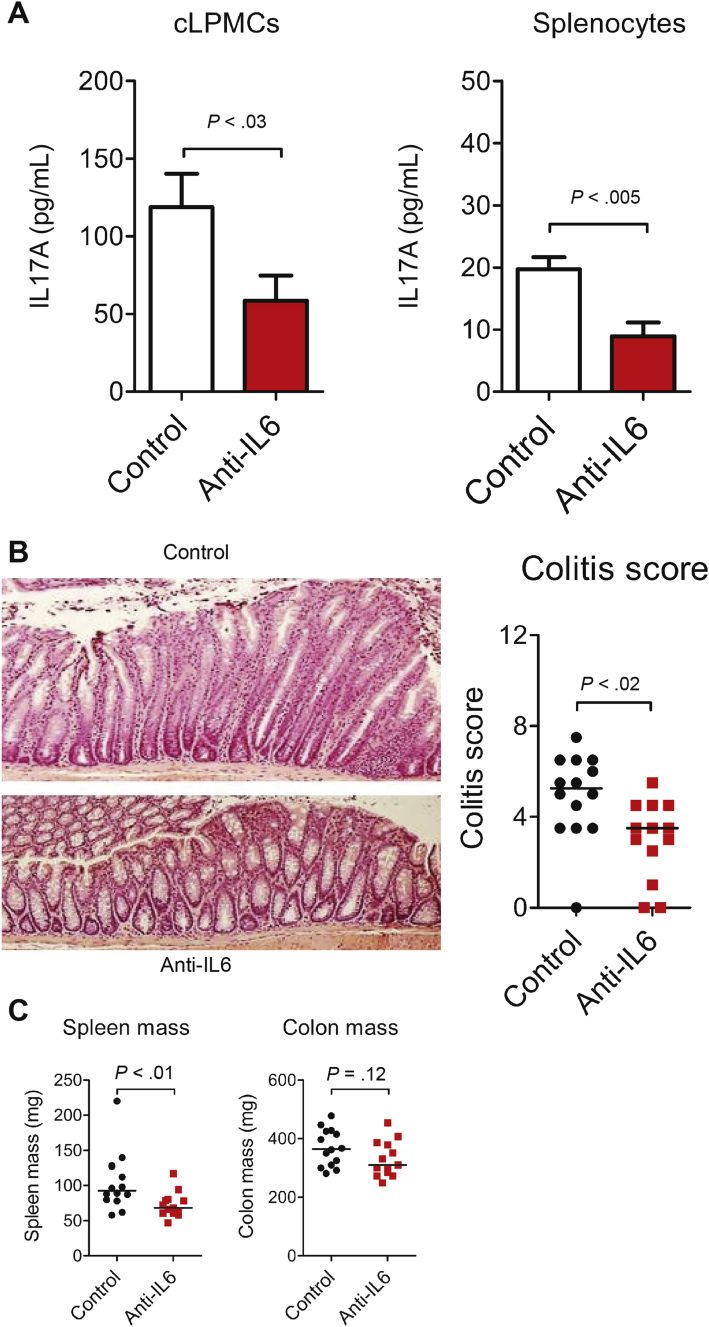
IL6 blockade reduces IL17A production and attenuates TRUC disease. (*A*) IL17A concentration in culture supernatants of unfractionated cLPMCs and splenocytes from TRUC mice treated with anti-IL6 (n = 8) or isotype-matched control antibodies (n = 8). (*B*) Representative colon micrographs (H&E stained) (*left panel*) and statistical analysis (*right panel*) of colitis scores of distal colons of TRUC mice treated with anti-IL6 or isotype-matched control antibodies. (*C*) Spleen and colon mass of TRUC mice treated with anti-IL6 or isotype-matched control antibodies. Each *dot/square* represents an individual mouse. *Lines* represent medians. Results from 2 separate antibody blockade experiments conducted under the same experimental conditions were pooled.

**Supplementary Figure 5:**
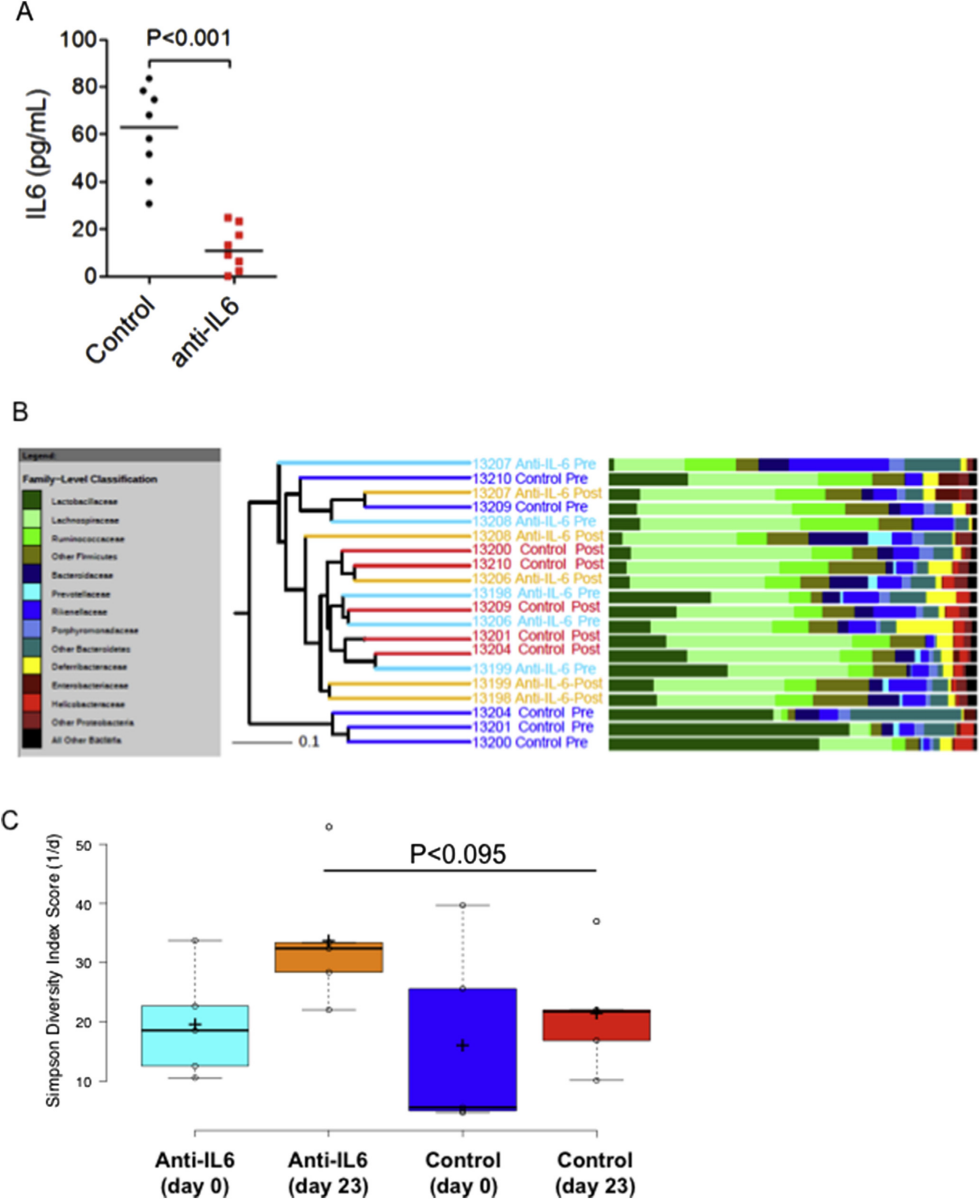
(*A*) Concentration of IL6 in serum of TRUC mice treated with anti-IL6 or control isotype antibody. *Dots/squares* represent individual mice. *Line* depicts median. (*B*) Bray Curtis cluster dendrogram showing that anti-IL6 treatment is not associated with a distinct microbiota profile. “Pre” indicates samples before anti-IL6 treatment and “Post” indicates samples after treatment. Bacterial families colored in shades of green belong to the Firmicutes phylum, blue belong to the Bacteroidetes phylum, yellow belong to the Deferribacteres phylum (*Mucispirillum* genus), and maroon/red belong to the Proteobacteria phylum. (*C*) *Box* and *whisker plots* of Simpson diversity index of the intestinal microbiota from TRUC mice treated with anti-IL6 or isotype-matched control antibodies. Diversity indices are highly sensitive to differential sequencing depth. Therefore, analyses were confined to 477 reads per sample.

To determine whether IL6-mediated activation of innate immunity was functionally important in TRUC disease, mice were treated with monoclonal antibodies that neutralize the biological activity of IL6. Treatment with anti-IL6 resulted in loss of IL6 bioavailability (Supplementary Figure 5*A*). IL17A production by unfractionated cLPMCs and splenocytes was reduced significantly in anti-IL6–treated TRUC mice, although was not abolished completely (Figure 3*A*). IL6 neutralization significantly attenuated TRUC disease, including reduced colitis scores and reduced splenomegaly (Figure 3*B* and *C*).

**Figure 4:**
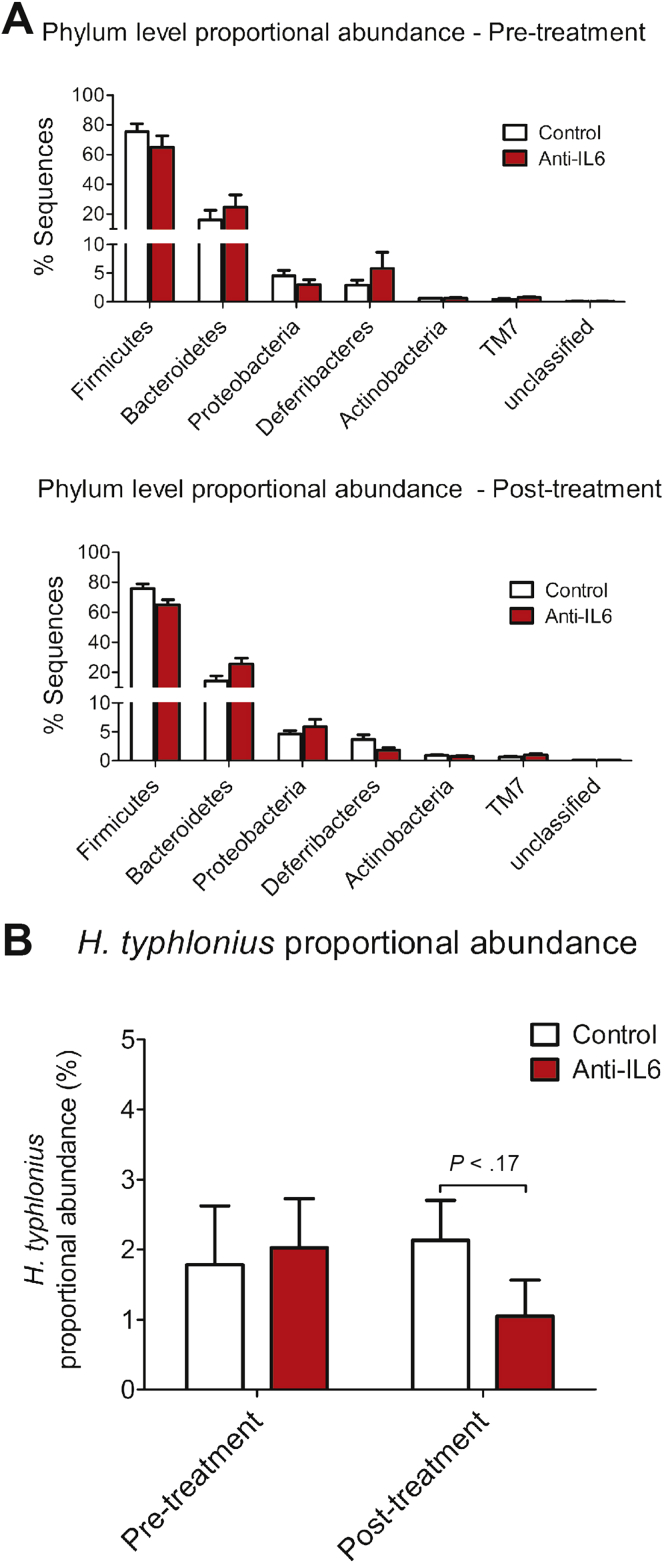
IL6 blockade does not significantly impact the composition of the intestinal microbiota in TRUC mice. (*A*) The mean percentage of sequences of particular phyla present in the intestinal microbiota of TRUC mice before (*top panel*) and after (*bottom panel*) treatment with anti-IL6 (*red bars*) or isotype-matched control antibodies (*white bars*). (*B*) The mean proportional abundance of *H typhlonius* in the intestinal microbiota of TRUC mice before and after treatment with anti-IL6 (*red bars*) or isotype-matched control antibodies (*white bars*). Error bars depict the SEM.

Similar to the situation in human IBD, TRUC disease is associated with perturbation of intestinal microbial communities. Because IL6 directly influences the success of mucosal colonization by some intestinal bacteria,^21^ we considered the possibility that attenuation of chronic TRUC disease after IL6 blockade might have occurred secondarily to changes in key components in the composition of the intestinal microbiota. To address this question we sequenced 16S ribosomal RNA genes that were PCR-amplified from fecal samples from anti-IL6 or control antibody–treated TRUC mice. Overall, we identified 2642 different operational taxonomic units (OTUs). Treatment with anti-IL6 antibody appeared to have a relatively minor impact on the microbiota (Figure 4*A*). At the phylum level, Firmicutes were reduced slightly in proportional abundance in anti-IL6–treated mice (*P* = .035) (Figure 4*A*) but, at finer taxonomic levels, anti-IL6 treatment did not impact the proportional abundance of the most common 150 OTUs significantly, which cumulatively accounted for more than 96% of the total amount of sequence data generated (Supplementary Table 2). Cluster analysis, using the Bray Curtis calculator, confirmed that there was no signature microbiota profile associated with anti-IL6 treatment (Supplementary Figure 5*B*). *Helicobacter typhlonius* was ubiquitously present in anti-IL6– or isotype control–treated mice, however, the proportional abundance did not differ significantly between the 2 groups either before or after treatment (Figure 4*B*). There was a tendency for increased bacterial diversity in the gut of anti-IL6–treated mice in comparison with control antibody–treated mice, although this did not achieve statistical significance (*P* < .095) (Supplementary Figure 5*C*).

### IL6 Augments Pathogenic Cytokine Production by Colonic CD3^-^ IL7R^+^ Cells From IBD Patients

**Figure 5:**
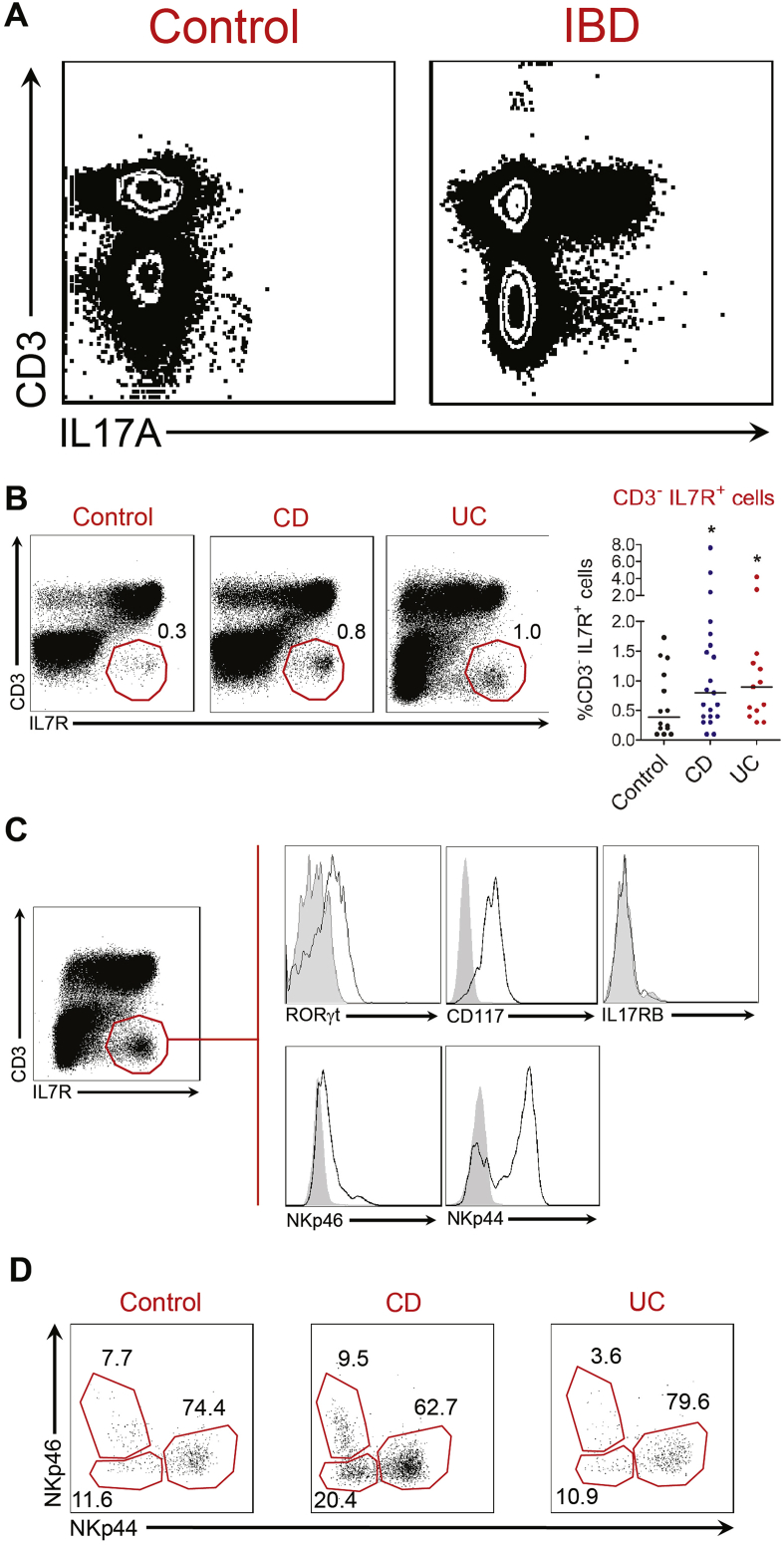
CD3^-^ IL7R^+^ cells are expanded in IBD patients. (*A*) Flow cytometry plots showing CD3 and intracellular IL17A expression in unfractionated cLPMCs after stimulation with PMA and ionomycin. Additional representative flow cytometry plots are illustrated in Supplementary Figure 6*A*. (*B*) Representative flow cytometry plots (*left panel*) and statistical analysis (*right panel*) of CD3 and IL7R staining by LPMCs in the colon of noninflammatory control and IBD patients. Individual *dots* represent individual patients. *P < .04. (*C*) Flow cytometric analysis of the phenotype of colonic CD3^-^ IL7R^+^ cells. *Grey histograms* show isotype control antibody staining. *White histograms* show staining with specific antibody. Data are representative of more than 3 independent experiments in IBD patients. (*D*) Flow cytometry dot plots showing expression of NKp46 and NKp44 by colonic CD3^-^ IL7R^+^ cells in noninflammatory control and IBD patients. Further analyses of additional patient replicates are shown in Supplementary Figure 6*C* and *D*.

**Supplementary Figure 6:**
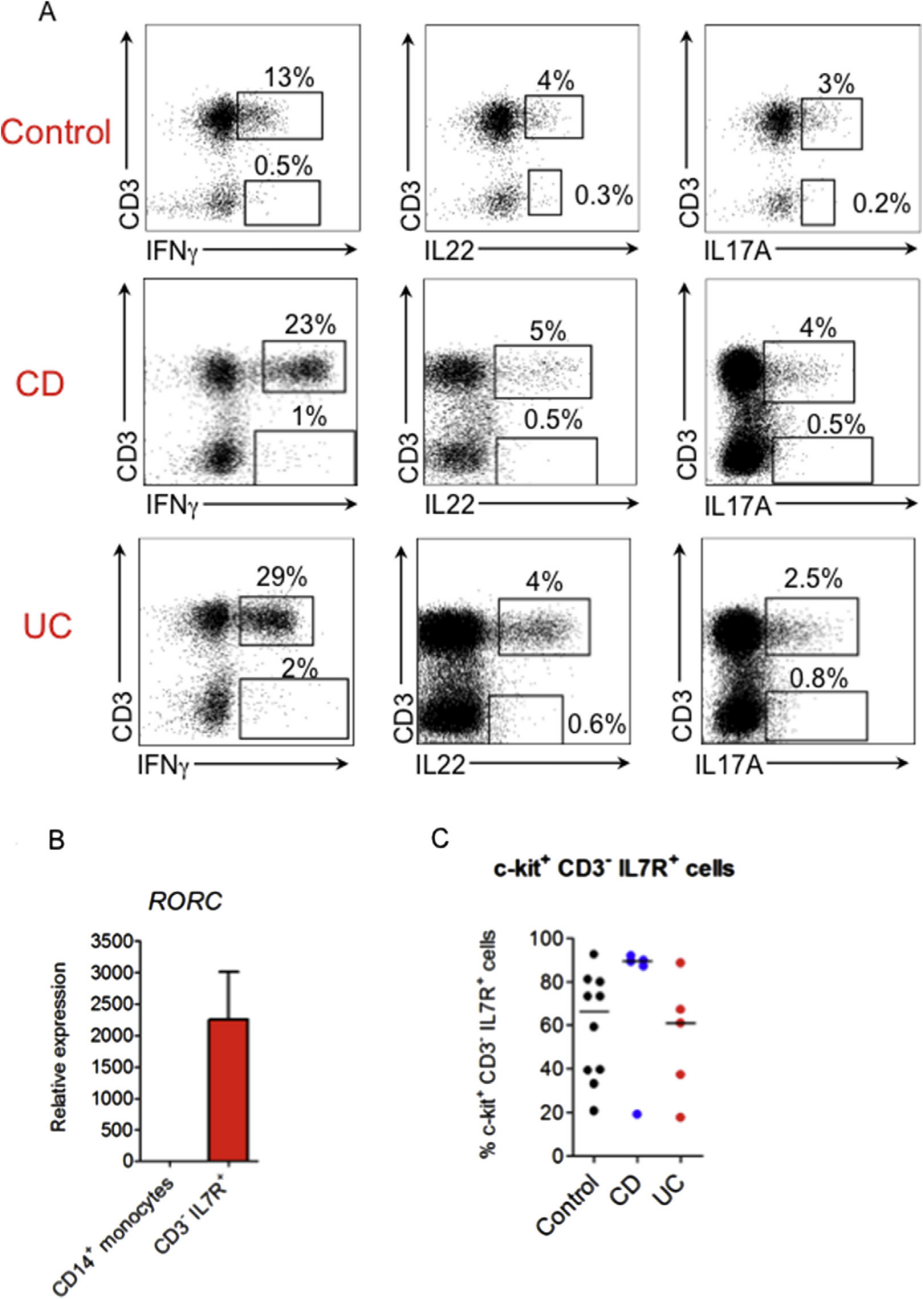

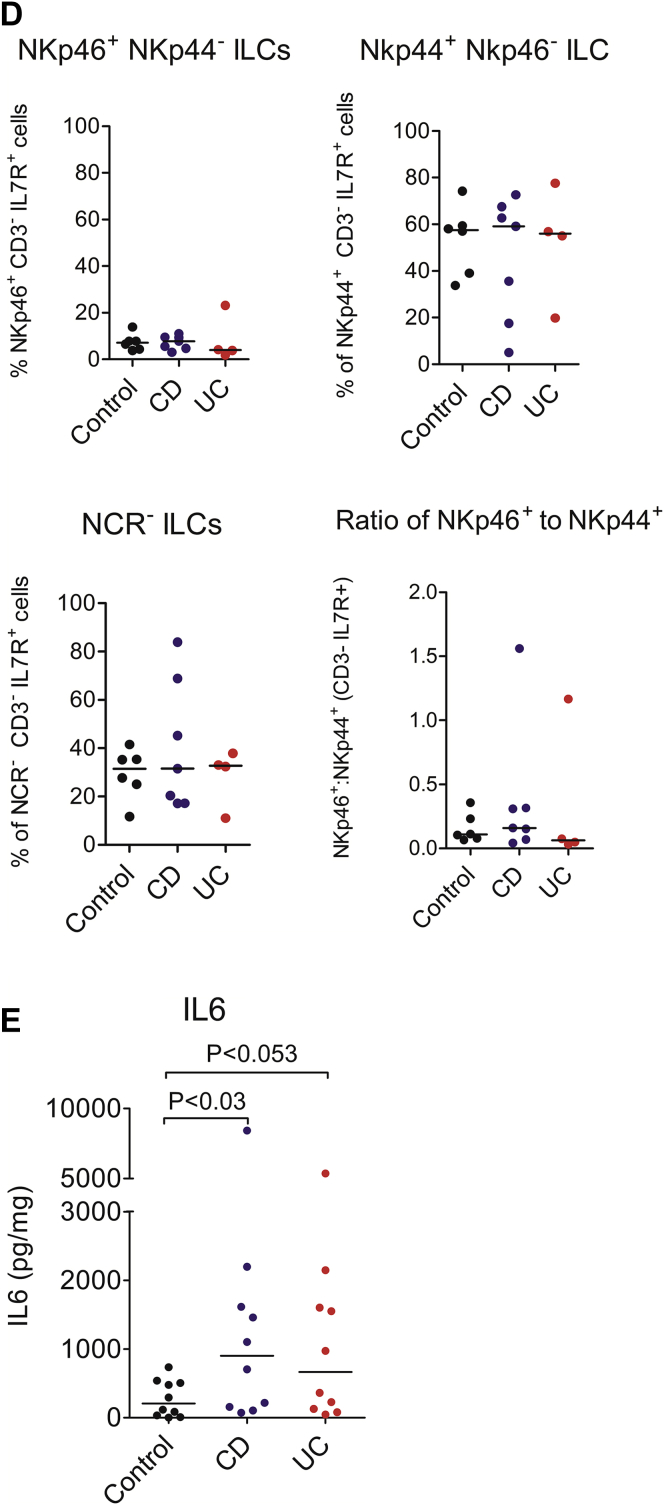
(*A*) Representative flow cytometry plots of intracellular cytokine and surface CD3 expression in noninflammatory control, CD, and UC patients. Cells were stimulated with PMA and ionomycin. (*B*) Real-time PCR analysis of *RORC* expression in sorted colonic CD3^-^ IL7R^+^ cells in comparison with CD14^+^ monocytes (immunomagnetically selected from peripheral blood monocytes). Histogram shows the mean expression of *RORC* in purified colonic CD3^-^ IL7R^+^ cells (n = 2 IBD patients) relative to monocytes. (*C*) Proportion of colonic CD3^-^ IL7R^+^ cells expressing c-kit (CD117) in noninflammatory control, CD, and UC patients. (*D*) Proportion of colonic CD3^-^ IL7R^+^ cells expressing NKp46, NKp44, or double-negative cells (NKp46^-^ NKp44^-^) in noninflammatory control, CD, and UC patients. (*E*) IL6 production in colon explant cultures from IBD patients. In graphs, each *dot* represents an individual patient and a *line* depicts the median.

Our preclinical data support the possibility that targeting IL6 may be therapeutically tractable in chronic gut inflammation. Therefore, we aimed to verify whether this pathway was relevant in human disease. As expected, stimulation of unfractionated intestinal immune cells with PMA and ionomycin resulted in production of pathogenic cytokines, including IL17A, IL22, and interferon-γ by CD3^+^ T cells (Figure 5*A* and Supplementary Figure 6*A*). However, we also observed production of these cytokines in the non–T-cell (CD3^-^) fraction, particularly in IBD patients. Within the non–T-cell fraction (CD3^-^), we could identify a population of IL7R-expressing cells in the colon of patients with CD, UC, and noninflammatory control patients (Figure 5*B*). Although the frequency of these cells was variable, their proportional abundance within the lymphocyte population was increased in IBD patients in comparison with noninflammatory controls (Figure 5*B*). Consistent with ILC3s being present among the CD3^-^ IL7R^+^ population, there was enriched expression of RORγt and c-kit (CD117) (Figure 5*C* and Supplementary Figure 6*C*). *RORC* transcripts also were enriched in fluorescence-activated cell sorter–purified CD3^-^ IL7R^+^ cells analyzed by real-time PCR (Supplementary Figure 6*B*), corroborating the likelihood of ILCs being present in the CD3^-^ IL7R^+^ population. Analysis of the CD3^-^ IL7R^+^ population according to NCR expression showed the presence of 3 discrete populations, comprising NKp46^+^ NKp44^-^ cells, NKp44^+^ NKp46^-^ cells, and NCR^-^ (NKp44^-^ NKp46^-^) cells (Figure 5*D* and Supplementary Figure 6*C*), indicating that NCR^+^ and NCR^-^ ILCs are present within this CD3^-^ IL7R^+^ population. In most patients, including IBD and noninflammatory control patients, CD3^-^ IL7R^+^ NKp44^+^ NKp46^-^ cells were the predominant subset present (Figure 5*D* and Supplementary Figure 6*C*).

**Figure 6:**
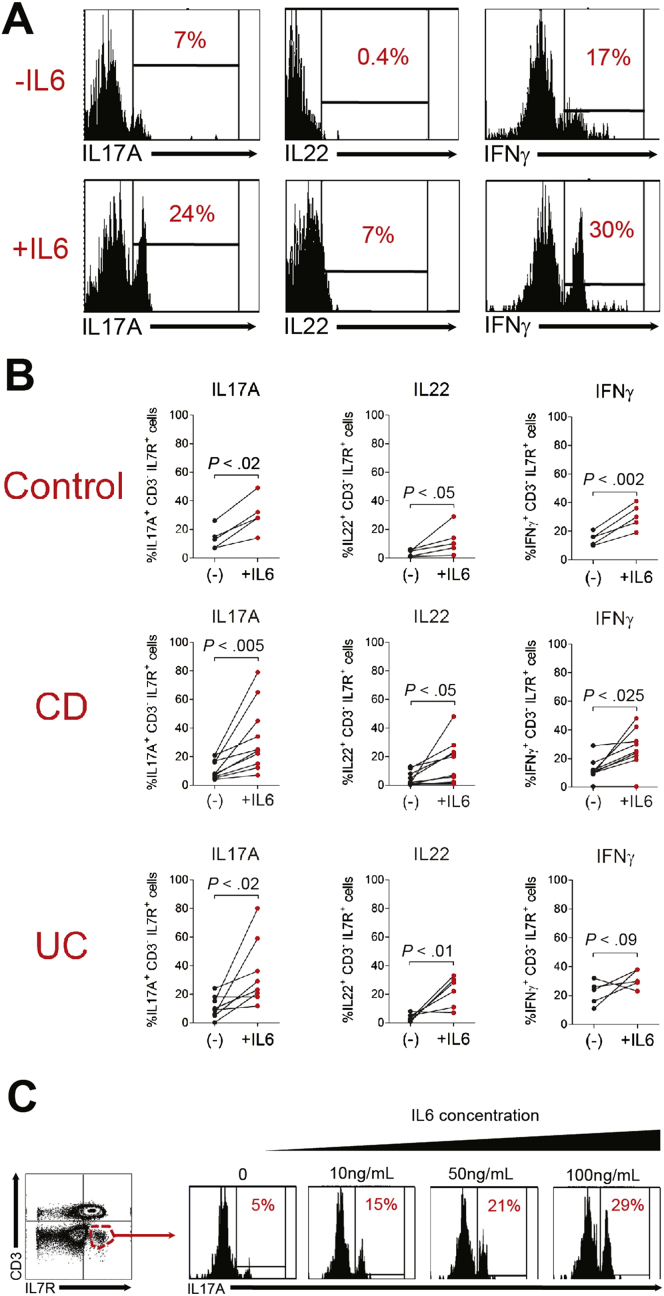
IL6-responsive CD3^-^ IL7R^+^ cells are present in the colon of IBD patients. (*A*) Flow cytometry histograms and (*B*) statistical analyses of intracellular cytokine expression by CD3^-^ IL7R^+^ cells after overnight culture in the presence or absence of IL6 (100 ng/mL). Cells were restimulated with PMA and ionomycin before staining. (*B*) Each connected pair of *dots* represents an individual patient. (*C*) Flow cytometry histograms showing the number of CD3^-^ IL7R^+^ IL17A^+^ cells after culture with increasing doses of IL6.

To determine whether CD3^-^ IL7R^+^ cells present in diseased mucosa of IBD patients were responsive to IL6, cLPMCs were incubated overnight with recombinant human IL6 before being restimulated with PMA and ionomycin. Production of IL17A, IL22, and interferon-γ by CD3^-^ IL7R^+^ cells was increased significantly when cLPMCs were cultured in the presence of IL6 (Figure 6*A* and *B*). In addition, some samples were stimulated with IL6 directly without mitogen, which showed induction of IL17A by CD3^-^ IL7R^+^ cells in a dose-dependent manner (Figure 6*C*).

Finally, we analyzed IL6 production by diseased mucosa from IBD patients to see whether blocking IL6 might be a reasonable therapeutic strategy in some or all IBD patients. IL6 was produced by colon explant cultures in CD, UC, and noninflammatory control patients (Supplementary Figure 6*D*). However, IL6 production was variable, especially in IBD patients, ranging from 72.2 to 8426.4 pg/mg colonic tissue. IBD patients could be stratified according to mucosal production of IL6, with half of IBD patients producing relatively low levels comparable with noninflammatory control patients and the other half producing high amounts (>1000 pg/mg tissue). Taken together, these data indicate that IL6, which is produced in very high quantities in approximately 50% of CD and UC patients drives pathologically relevant immune pathways in chronic intestinal inflammation.

## Discussion

CD4^-^ NCR^-^ ILC3s are the predominant CD90^+^ IL7R^+^ ILC population in the colon of TRUC mice responsible for causing disease. Purified intestinal NCR^-^ ILC3 from TRUC mice produced IL17A and IL22, but were poor producers of interferon-γ and tumor necrosis factor α. They were also a modest source of IL6. Few NCR^+^ ILCs were present in the colon of TRUC mice, consistent with data from other groups reporting a requirement for T-bet in NKp46^+^ ILC development and differentiation.^22^ Intestinal CD4^+^ ILCs are important in host resistance to intestinal pathogens, such as *Citrobacter rodentium*.^5^ Here, we show that CD4^+^ ILCs, which are abundant in mLN, but infrequent in the colon, do not play a major role in TRUC disease. Depletion of CD4^+^ ILCs had no impact on pathogenic cytokine production or disease outcome.

In TRUC mice, highly purified colonic NCR^-^ ILC3 did not respond to IL23 or IL1α in isolation. Instead, combinations of IL23 together with IL1α were required for production of effector cytokines by ILC3. Furthermore, additional exposure to IL6 was required for optimal IL17A and IL22 production, showing a novel role for IL6 in the innate immune system in chronic intestinal inflammation.

IL17A-, IL22-, and interferon-γ–producing CD3^-^ IL7R^+^ cells also were identified in the colonic lamina propria of patients with IBD. Although this population is heterogeneous, there was enrichment of RORγt and c-kit, confirming the likelihood that ILC3 were present within this compartment. Most CD3^-^ IL7R^+^ cells were NKp44^+^, although NKp46^+^ and NCR^-^ (NKp44^-^ NKp46^-^) cells also were present. These data are broadly consistent with previous reports of ILC populations in human gut.^12^, ^13^, ^14^ Crucially, IL6 increased pathogenic cytokine production by CD3^-^ IL7R^+^ cLPMCs from IBD patients in a dose-dependent manner, consistent with our preclinical data showing IL6-responsive colonic ILC3s.

IL6 is a pleiotropic cytokine that may be important in IBD. Peripheral blood and cLPMCs produce excess IL6 in IBD,^23^, ^24^ often at levels correlating with disease activity.^25^ Genetic variation at the *IL6* locus is linked with early onset IBD^26^ and polymorphisms at loci encoding IL6R signaling components are associated with increased IBD risk.^3^ IL6 blockade is therapeutic in some preclinical models of IBD, although it has been assumed that the therapeutic mechanism likely was attributable to limitation of T-cell–mediated pathology^27^, ^28^, ^29^, ^30^ because IL6 contributes to intestinal Th17 differentiation.^31^ We show a novel role of IL6 in innate immune-mediated chronic intestinal pathology. It is interesting that cytokines contributing to CD4^+^ Th17 differentiation, including IL1, IL23, and IL6, have conserved roles promoting innate IL17 production. Our data build on other work implicating ILCs as potentially important mediators in IBD,^12^, ^13^, ^14^ and confirm NCR^-^ ILC3 as a source of pathogenic cytokines in IBD. Polymorphisms at multiple susceptibility loci in IBD that previously were considered to impact adaptive immunity, similarly could impact ILC phenotype, including *RORC*, *IL23R*, *IL12RB2*, *IL12B*, *IL22*, *IFNG*, *STAT1*, *STAT3*, *STAT4*, *CCR6*, *IL1R1*, *IL15RA*, and *IL6ST*.^3^ Accordingly, it is possible that genetic variation at these loci in IBD could impact disease susceptibility by altering the activation and effector function of mucosal ILCs. However, the relative contribution of ILC to the initiation and propagation of chronic intestinal inflammation in IBD remains to be determined. Polyclonal stimulus of unfractionated cLPMCs from IBD patients showed that most cytokine-expressing cells reside within the CD3^+^ cell fraction. It should be remembered that cytokine responses induced by polyclonal stimuli may overestimate T-cell contribution because under physiological conditions few of these tissue-trafficking T cells would be encountering their relevant antigen, so would unlikely be triggered to produce cytokine. By contrast, despite their numeric inferiority to T cells, mucosal ILC are likely to be activated directly by cytokine signals abundant in chronically inflamed tissue, such as IL6. IL6-induced stimulation of ILC effector function may prove to be especially pertinent in UC because IL23, the canonical ILC-activating cytokine, is produced at low levels in UC in comparison with CD.^32^

In this study, IL6 neutralization reduced innate production of IL17A in TRUC mice and significantly attenuated disease severity, although the magnitude of impact was less than seen with ILC depletion or IL23 blockade.^7^ This is in keeping with our observation that although IL6 is required for optimal activation of ILC effector function, other proximal cytokine signals, including IL23 and/or IL1 stimulation, additionally are required. IL6 blockade had a minimal impact on the intestinal microbiota, other than a minor shift in the proportional abundance of Firmicutes and a tendency for increased intestinal bacterial diversity. It is possible that this latter change occurred secondary to reduced intestinal inflammation in anti-IL6–treated mice. Indeed, IBD activity/severity is recognized to correlate inversely with bacterial diversity in the gut.^33^, ^34^

Our data support extending biological therapies targeting IL6 in IBD. The IL6R blocking antibody tocilizumab is efficacious in other inflammatory diseases, including arthritis^35^, ^36^ and lupus,^37^ and a pilot study in CD showed promising initial results.^38^ In this study mucosal IL6 production was highly variable, however, only half of IBD patients produced more IL6 than non-IBD control patients. Similarly, the frequency of CD3^-^ IL7R^+^ cells, and the magnitude of IL6-induced cytokine responses by these cells also markedly was variable. With the promise of personalized medicine on the horizon,^39^ it is tempting to speculate that treatment strategies targeting IL6 might be favored in patient subsets defined by high mucosal expression of IL6 and/or high frequencies of IL6 responsive effector cells in diseased tissue.

In summary, we have shown that IL6 augments pathogenic cytokine production by intestinal ILCs in chronic intestinal inflammation and that this pathway may be operational in human IBD. Novel therapeutic strategies targeting ILC or their proximal cytokine signals may offer a new treatment paradigm in IBD.

## Figures and Tables

**Supplementary Table 1:** Flow Cytometry Antibodies Used

Antigen	Clone	Supplier
Anti-mouse antibodies		
CD45	30-F11	eBioscience
CD90.2	53-2.1	eBioscience
CD127	A7R34	eBioscience
NKp46	29A1.4	eBioscience
CD126	D7715A7	Biolegend
CCR6	140706	R&D Systems
ICOS	7E.17G9	eBioscience
CD4	RM4.5	eBioscience
Hematopoietic lineage cocktail (CD3, CD45R, B220, CD11b, TER-119, Gr-1)	17A2, RA3-6B2, M1/70, TER-119	eBioscience
CD62L	MEL-14	eBioscience
IL17RB	752101	R&D Systems
CD69	H1.2F3	eBioscience
RORγt	AFKJS-9	eBioscience
IL17A	eBio17B7	eBioscience
IL22	1H8PWSR	eBioscience
Interferon-γ	XMG1.2	eBioscience
IL4	11B11	eBioscience
Anti-human antibodies		
CD3	UCHT-1	Biolegend
CD127	A019D5 (eBioRDR5)	Biolegend (eBioscience)
CD117	104D2	eBioscience
NKp46	9E2	eBioscience
NKp44	44.189	eBioscience
IL17RB	I170220	R&D Systems
RORγt	AFKJS-9	eBioscience
IL17A	eBio64DEC17	eBioscience
IL22	22URTI	eBioscience
Interferon-γ	4S.B3	eBioscience

**Supplementary Table 2:** Metastats Comparison Between the Proportional Abundance of the Top 150 Most Abundant OTUs in the Microbiota Data Set Between Anti-IL6 and Control Mice Groups

OTU no.	RDP taxonomic classifications					NCBI MegaBLAST ID	*P* value	Q value
	Phylum	Class	Order	Family	Genus			
Otu0001	Firmicutes(100)	Bacilli(100)	Lactobacillales(100)	Lactobacillaceae(100)	Lactobacillus(100)	*Lactobacillus animalis*/*murinus*	.287595	1
Otu0002	Firmicutes(100)	Clostridia(100)	Clostridiales(100)	Lachnospiraceae(100)	unclassified(100)	*Butyrivibrio* species P79 (86% similarity)	.022376	1
Otu0003	Firmicutes(100)	Bacilli(100)	Lactobacillales(100)	Lactobacillaceae(100)	Lactobacillus(100)	*Lactobacillus taiwanensis*/*johnsonii*/*acidophilus*	.947484	1
Otu0004	Firmicutes(100)	Clostridia(100)	Clostridiales(100)	Lachnospiraceae(100)	Clostridium_XlVb(100)	*Clostridium lactatifermentans* (90% similarity)	.15343	1
Otu0005	Firmicutes(100)	Clostridia(100)	Clostridiales(100)	Lachnospiraceae(100)	Unclassified(100)	*Eubacterium plexicaudatum* (92% similarity)	.82004	1
Otu0006	Bacteroidetes(100)	Bacteroidia(100)	Bacteroidales(100)	Unclassified(100)	Unclassified(100)	*Butyricimonas* species JCM 18677 (80% similarity)	.012182	1
Otu0007	Proteobacteria(100)	Epsilonproteobacteria(100)	Campylobacterales(100)	Helicobacteraceae(100)	Helicobacter(100)	*Helicobacter typhlonius*	.173889	1
Otu0008	Deferribacteres(100)	Deferribacteres(100)	Deferribacterales(100)	Deferribacteraceae(100)	Mucispirillum(100)	*Mucispirillum colimuris*	.063365	1
Otu0009	Bacteroidetes(100)	Bacteroidia(100)	Bacteroidales(100)	Rikenellaceae(100)	Alistipes(100)	*Alistipes onderdonkii* (90% similarity)	.874014	1
Otu0010	Bacteroidetes(100)	Bacteroidia(100)	Bacteroidales(100)	Rikenellaceae(100)	Alistipes(100)	*Alistipes senegalensis* (91% similarity)	.800781	1
Otu0011	Firmicutes(100)	Clostridia(100)	Clostridiales(100)	Unclassified(100)	Unclassified(100)	*Clostridium scindens* (88% similarity)	.766219	1
Otu0012	Firmicutes(100)	Clostridia(100)	Clostridiales(100)	Lachnospiraceae(100)	Unclassified(100)	*Coprococcus catus* (88% similarity)	.571687	1
Otu0013	Firmicutes(100)	Clostridia(100)	Clostridiales(100)	Ruminococcaceae(100)	Oscillibacter(100)	*Oscillibacter valericigenes* (91% similarity)	.03155	1
Otu0014	Bacteroidetes(100)	Bacteroidia(100)	Bacteroidales(100)	Bacteroidaceae(100)	Bacteroides(100)	*Bacteroides acidofaciens*/*uniformis*	.082054	1
Otu0015	Firmicutes(100)	Clostridia(100)	Clostridiales(100)	Ruminococcaceae(100)	Anaerotruncus(100)	*Anaerotruncus colihominis* (92% similarity)	.489023	1
Otu0016	Firmicutes(100)	Clostridia(100)	Clostridiales(100)	Lachnospiraceae(100)	Unclassified(100)	*Clostridium hathewayi* (92% similarity)	.861923	1
Otu0017	Firmicutes(100)	Bacilli(100)	Lactobacillales(100)	Lactobacillaceae(100)	Lactobacillus(100)	*Lactobacillus reuteri*	.644746	1
Otu0018	Proteobacteria(100)	Deltaproteobacteria(100)	Unclassified(100)	Unclassified(100)	Unclassified(100)	*Desulfocurvus vexinensis* (87% similarity)	.44467	1
Otu0019	Deferribacteres(100)	Deferribacteres(100)	Deferribacterales(100)	Deferribacteraceae(100)	Mucispirillum(100)	*Mucispirillum colimuris* (97% similarity)	.039672	1
Otu0020	Bacteroidetes(100)	Bacteroidia(100)	Bacteroidales(100)	Unclassified(99)	Unclassified(99)	*Tannerella forsythensis* (82% similarity)	.262652	1
Otu0021	Bacteroidetes(100)	Bacteroidia(100)	Bacteroidales(100)	Bacteroidaceae(100)	Bacteroides(100)	*Bacteroides acidifaciens*	.733786	1
Otu0022	Bacteroidetes(100)	Bacteroidia(100)	Bacteroidales(100)	Prevotellaceae(100)	Paraprevotella(100)	*Prevotella species* (87% similarity)	.137208	1
Otu0023	Firmicutes(100)	Clostridia(100)	Clostridiales(100)	Lachnospiraceae(52)	Unclassified(52)	*Coprococcus catus* (84% similarity)	.501202	1
Otu0024	Firmicutes(100)	Clostridia(100)	Clostridiales(100)	Lachnospiraceae(100)	Unclassified(100)	*Pseudobutyrivibrio ruminis* (88% similarity)	.510185	1
Otu0025	Bacteroidetes(100)	Bacteroidia(100)	Bacteroidales(100)	Porphyromonadaceae(100)	Paludibacter(77)	*Prevotella dentalis* (81% similarity)	.162484	1
Otu0026	Bacteroidetes(100)	Bacteroidia(100)	Bacteroidales(100)	Bacteroidaceae(100)	Bacteroides(100)	*Bacteroides uniformis* (96% similarity)	.195279	1
Otu0027	Firmicutes(100)	Erysipelotrichia(99)	Erysipelotrichales(99)	Erysipelotrichaceae(99)	Erysipelotrichaceae_incertae_sedis(99)	*Eubacterium cylindroides* (88% similarity)	.605205	1
Otu0028	Firmicutes(100)	Clostridia(100)	Clostridiales(100)	Unclassified(100)	Unclassified(100)	*Clostridium aminophilum* (86% similarity)	.246769	1
Otu0029	TM7(100)	TM7_class_incertae_sedis(100)	TM7_order_incertae_sedis(100)	TM7_family_incertae_sedis(100)	TM7_genus_incertae_sedis(100)	TM7 phylum species (93% similarity)	.222701	1
Otu0030	Firmicutes(100)	Clostridia(100)	Clostridiales(100)	Lachnospiraceae(100)	Unclassified(100)	*Clostridium phytofermentans* (90% similarity)	.574396	1
Otu0031	Firmicutes(100)	Clostridia(100)	Clostridiales(100)	Lachnospiraceae(100)	Unclassified(100)	*Clostridium celerecrescens* (93% similarity)	.664006	1
Otu0032	Firmicutes(100)	Clostridia(100)	Clostridiales(100)	Unclassified(100)	Unclassified(100)	*Clostridium scindens* (84% similarity)	.737231	1
Otu0033	Proteobacteria(100)	Gammaproteobacteria(100)	Enterobacteriales(100)	Enterobacteriaceae(100)	Unclassified	*Escherichia*/*Enterobacter*/*Citrobacter*/*Shigella* species	.497501	1
Otu0034	Bacteroidetes(100)	Bacteroidia(100)	Bacteroidales(100)	Rikenellaceae(100)	Alistipes(100)	*Alistipes senegalensis* (92% similarity)	.127302	1
Otu0035	Firmicutes(100)	Unclassified(100)	Unclassified(100)	Unclassified(100)	Unclassified(100)	Segmented filamentous bacterium	.798342	1
Otu0036	Firmicutes(100)	Clostridia(100)	Clostridiales(100)	Lachnospiraceae(100)	Unclassified(100)	*Eubacterium oxidoreducens* (87%)	.334068	1
Otu0037	Firmicutes(100)	Clostridia(100)	Clostridiales(100)	Ruminococcaceae(100)	Oscillibacter(100)		.263709	1
Otu0038	Bacteroidetes(100)	Bacteroidia(100)	Bacteroidales(100)	Unclassified(100)	Unclassified(100)		.951701	1
Otu0039	Bacteroidetes(100)	Bacteroidia(100)	Bacteroidales(100)	Bacteroidaceae(100)	Bacteroides(100)		.178105	1
Otu0040	Firmicutes(100)	Clostridia(100)	Clostridiales(100)	Lachnospiraceae(100)	Lachnospiracea_incertae_sedis(79)		.987598	1
Otu0041	Firmicutes(100)	Clostridia(100)	Clostridiales(100)	Ruminococcaceae(100)	Oscillibacter(100)		.948299	1
Otu0042	Bacteroidetes(100)	Bacteroidia(100)	Bacteroidales(100)	Bacteroidaceae(100)	Bacteroides(100)		.334148	1
Otu0043	Firmicutes(100)	Clostridia(100)	Clostridiales(100)	Lachnospiraceae(100)	Dorea(93)		.850598	1
Otu0044	Firmicutes(100)	Clostridia(100)	Clostridiales(100)	Ruminococcaceae(100)	Anaerotruncus(100)		.622208	1
Otu0045	Firmicutes(100)	Clostridia(100)	Clostridiales(100)	Lachnospiraceae(100)	Unclassified(100)		.948849	1
Otu0046	Bacteroidetes(100)	Bacteroidia(100)	Bacteroidales(100)	Rikenellaceae(100)	Alistipes(100)		.211071	1
Otu0047	Firmicutes(100)	Clostridia(100)	Clostridiales(100)	Lachnospiraceae(100)	Clostridium_XlVb(100)		.449205	1
Otu0048	Firmicutes(100)	Clostridia(100)	Clostridiales(100)	Unclassified(100)	Unclassified(100)		.101219	1
Otu0049	Firmicutes(100)	Clostridia(100)	Clostridiales(100)	Ruminococcaceae(100)	Butyricicoccus(100)		.597761	1
Otu0050	Bacteroidetes(100)	Bacteroidia(100)	Bacteroidales(100)	Porphyromonadaceae(100)	Odoribacter(100)		.824846	1
Otu0051	Firmicutes(100)	Clostridia(100)	Clostridiales(100)	Lachnospiraceae(100)	Unclassified(72)		.889006	1
Otu0052	Bacteroidetes(100)	Unclassified(99)	Unclassified(99)	Unclassified(99)	Unclassified(99)		.352835	1
Otu0053	Firmicutes(100)	Clostridia(100)	Clostridiales(100)	Lachnospiraceae(100)	Unclassified(100)		.420749	1
Otu0054	Firmicutes(100)	Clostridia(100)	Clostridiales(100)	Ruminococcaceae(100)	Oscillibacter(100)		.807154	1
Otu0055	Firmicutes(100)	Clostridia(100)	Clostridiales(100)	Lachnospiraceae(100)	Roseburia(100)		.549208	1
Otu0056	Firmicutes(100)	Clostridia(100)	Clostridiales(100)	Lachnospiraceae(100)	unclassified(100)		.795695	1
Otu0057	Firmicutes(100)	Clostridia(100)	Clostridiales(100)	Ruminococcaceae(100)	Acetanaerobacterium(100)		.795544	1
Otu0058	Firmicutes(100)	Clostridia(100)	Clostridiales(100)	Lachnospiraceae(100)	Unclassified(100)		.594923	1
Otu0059	Firmicutes(100)	Clostridia(100)	Clostridiales(100)	Lachnospiraceae(100)	Unclassified(100)		.319484	1
Otu0060	Firmicutes(100)	Unclassified(100)	Unclassified(100)	Unclassified(100)	Unclassified(100)		.815476	1
Otu0061	Firmicutes(100)	Clostridia(100)	Clostridiales(100)	Unclassified(100)	Unclassified(100)		.245678	1
Otu0062	Firmicutes(100)	Clostridia(100)	Clostridiales(100)	Lachnospiraceae(100)	Roseburia(100)		.642752	1
Otu0063	Firmicutes(100)	Clostridia(100)	Clostridiales(100)	Lachnospiraceae(100)	Unclassified(100)		.866248	1
Otu0064	Firmicutes(100)	Clostridia(100)	Clostridiales(100)	Unclassified(100)	Unclassified(100)		.684305	1
Otu0065	Actinobacteria(100)	Actinobacteria(100)	Coriobacteriales(100)	Coriobacteriaceae(100)	Enterorhabdus(100)		.470026	1
Otu0066	Firmicutes(100)	Unclassified(100)	Unclassified(100)	Unclassified(100)	Unclassified(100)		.781442	1
Otu0067	Firmicutes(100)	Clostridia(100)	Clostridiales(100)	Lachnospiraceae(100)	Unclassified(100)		.075425	1
Otu0068	Firmicutes(100)	Clostridia(100)	Clostridiales(100)	Lachnospiraceae(100)	Unclassified(100)		.23604	1
Otu0069	Proteobacteria(100)	Deltaproteobacteria(100)	Desulfovibrionales(100)	Desulfovibrionaceae(100)	Desulfovibrio(93)		.122282	1
Otu0070	Firmicutes(100)	Clostridia(100)	Clostridiales(100)	Lachnospiraceae(100)	Dorea(100)		.335755	1
Otu0071	Firmicutes(100)	Unclassified(100)	Unclassified(100)	Unclassified(100)	Unclassified(100)		.275564	1
Otu0072	Bacteroidetes(100)	Bacteroidia(100)	Bacteroidales(100)	Porphyromonadaceae(100)	Unclassified(100)		.6759	1
Otu0073	Firmicutes(100)	Clostridia(100)	Clostridiales(100)	Ruminococcaceae(98)	Unclassified(98)		.739009	1
Otu0074	Firmicutes(100)	Clostridia(100)	Clostridiales(100)	Ruminococcaceae(100)	Oscillibacter(100)		.099638	1
Otu0075	Bacteroidetes(100)	Bacteroidia(100)	Bacteroidales(100)	Porphyromonadaceae(100)	Unclassified(100)		.142259	1
Otu0076	Firmicutes(100)	Clostridia(100)	Clostridiales(100)	Lachnospiraceae(100)	Unclassified(100)		.446379	1
Otu0077	Firmicutes(100)	Clostridia(100)	Clostridiales(100)	Lachnospiraceae(100)	Butyrivibrio(91)		.048125	1
Otu0078	Actinobacteria(100)	Actinobacteria(100)	Coriobacteriales(100)	Coriobacteriaceae(100)	Unclassified(100)		.020051	1
Otu0079	Firmicutes(100)	Clostridia(100)	Clostridiales(100)	Lachnospiraceae(100)	Unclassified(100)		.995336	1
Otu0080	Firmicutes(100)	Clostridia(100)	Clostridiales(100)	Lachnospiraceae(100)	Dorea(100)		.580909	1
Otu0081	Firmicutes(100)	Clostridia(100)	Clostridiales(100)	Lachnospiraceae(100)	Butyrivibrio(96)		.231131	1
Otu0082	Firmicutes(100)	Clostridia(100)	Clostridiales(100)	Lachnospiraceae(100)	Unclassified(100)		.144752	1
Otu0083	Actinobacteria(100)	Actinobacteria(100)	Coriobacteriales(100)	Coriobacteriaceae(100)	Asaccharobacter(100)		.683732	1
Otu0084	Firmicutes(100)	Clostridia(100)	Clostridiales(100)	Lachnospiraceae(100)	Unclassified(100)		.059598	1
Otu0085	Firmicutes(100)	Clostridia(100)	Clostridiales(100)	Ruminococcaceae(100)	Anaerotruncus(100)		.557724	1
Otu0086	Firmicutes(100)	Clostridia(100)	Clostridiales(100)	Ruminococcaceae(100)	Anaerotruncus(100)		.829296	1
Otu0087	Firmicutes(100)	Clostridia(100)	Clostridiales(100)	Ruminococcaceae(100)	Unclassified(100)		.147604	1
Otu0088	Firmicutes(100)	Clostridia(100)	Clostridiales(100)	Lachnospiraceae(100)	Unclassified(100)		.325188	1
Otu0089	Firmicutes(100)	Clostridia(100)	Clostridiales(100)	Lachnospiraceae(100)	Unclassified(100)		.270353	1
Otu0090	Firmicutes(100)	Clostridia(100)	Clostridiales(100)	Lachnospiraceae(100)	Clostridium_XlVa(98)		.893607	1
Otu0091	Bacteroidetes(100)	Bacteroidia(100)	Bacteroidales(100)	Unclassified(97)	Unclassified(97)		.064746	1
Otu0092	Firmicutes(100)	Clostridia(100)	Clostridiales(100)	Lachnospiraceae(100)	Syntrophococcus(100)		.465783	1
Otu0093	Proteobacteria(100)	Deltaproteobacteria(100)	Desulfovibrionales(100)	Desulfovibrionaceae(100)	Desulfocurvus(73)		.719197	1
Otu0094	Firmicutes(100)	Clostridia(100)	Clostridiales(100)	Ruminococcaceae(100)	Pseudoflavonifractor(100)		.223923	1
Otu0095	Firmicutes(100)	Clostridia(100)	Clostridiales(100)	Ruminococcaceae(100)	Pseudoflavonifractor(65)		.323615	1
Otu0096	Firmicutes(100)	Clostridia(100)	Clostridiales(100)	Lachnospiraceae(100)	Unclassified(100)		.220402	1
Otu0097	Firmicutes(100)	Clostridia(100)	Clostridiales(100)	Lachnospiraceae(100)	Unclassified(100)		.755524	1
Otu0098	Firmicutes(100)	Erysipelotrichia(100)	Erysipelotrichales(100)	Erysipelotrichaceae(100)	Clostridium_XVIII(100)		.167966	1
Otu0099	Firmicutes(100)	Clostridia(100)	Clostridiales(100)	Lachnospiraceae(100)	Unclassified(100)		.658952	1
Otu0100	Firmicutes(100)	Bacilli(100)	Lactobacillales(100)	Streptococcaceae(100)	Streptococcus(100)		.271556	1
Otu0101	Firmicutes(100)	Clostridia(100)	Clostridiales(100)	Ruminococcaceae(100)	Oscillibacter(100)		.529912	1
Otu0102	Firmicutes(100)	Clostridia(100)	Clostridiales(100)	Ruminococcaceae(100)	Unclassified(98)		.239692	1
Otu0103	Actinobacteria(100)	Actinobacteria(100)	Coriobacteriales(100)	Coriobacteriaceae(100)	Unclassified(100)		.438692	1
Otu0104	Firmicutes(100)	Clostridia(100)	Clostridiales(100)	Ruminococcaceae(100)	Flavonifractor(97)		.884299	1
Otu0105	Bacteroidetes(100)	Bacteroidia(100)	Bacteroidales(100)	Porphyromonadaceae(100)	Parabacteroides(100)		.604151	1
Otu0106	Firmicutes(100)	Clostridia(100)	Clostridiales(100)	Lachnospiraceae(100)	Unclassified(100)		.052835	1
Otu0107	Firmicutes(100)	Clostridia(100)	Clostridiales(100)	Lachnospiraceae(100)	Lactonifactor(100)		.688385	1
Otu0108	Firmicutes(100)	Unclassified(100)	Unclassified(100)	Unclassified(100)	Unclassified(100)		.808444	1
Otu0109	Unclassified(100)	Unclassified(100)	Unclassified(100)	Unclassified(100)	Unclassified(100)		.580963	1
Otu0110	Bacteroidetes(100)	Bacteroidia(100)	Bacteroidales(100)	Rikenellaceae(100)	Alistipes(100)		.590556	1
Otu0111	Firmicutes(100)	Clostridia(100)	Clostridiales(100)	Lachnospiraceae(100)	Unclassified(100)		.70702	1
Otu0112	Firmicutes(100)	Clostridia(100)	Clostridiales(100)	Ruminococcaceae(100)	Anaerotruncus(100)		.629385	1
Otu0113	Firmicutes(100)	Clostridia(100)	Clostridiales(100)	Lachnospiraceae(100)	Clostridium_XlVa(97)		.690915	1
Otu0114	Firmicutes(100)	Clostridia(100)	Clostridiales(100)	Ruminococcaceae(100)	Oscillibacter(100)		.659852	1
Otu0115	Firmicutes(100)	Clostridia(100)	Clostridiales(100)	Unclassified(100)	Unclassified(100)		.052071	1
Otu0116	Firmicutes(100)	Clostridia(100)	Clostridiales(100)	Ruminococcaceae(100)	Clostridium_IV(100)		.496749	1
Otu0117	Firmicutes(100)	Clostridia(100)	Clostridiales(100)	Lachnospiraceae(100)	Lachnospiracea_incertae_sedis(64)		.783083	1
Otu0118	Firmicutes(100)	Clostridia(100)	Clostridiales(100)	Lachnospiraceae(100)	Unclassified(100)		.450217	1
Otu0119	Firmicutes(100)	Clostridia(100)	Clostridiales(100)	Ruminococcaceae(100)	Anaerotruncus(100)		.474766	1
Otu0120	Firmicutes(100)	Clostridia(100)	Clostridiales(100)	Lachnospiraceae(100)	Roseburia(100)		.677578	1
Otu0121	Firmicutes(100)	Clostridia(100)	Clostridiales(100)	Ruminococcaceae(100)	Pseudoflavonifractor(100)		.764818	1
Otu0122	Firmicutes(100)	Clostridia(100)	Clostridiales(100)	Lachnospiraceae(100)	Roseburia(71)		.221575	1
Otu0123	Proteobacteria(100)	Deltaproteobacteria(100)	Desulfovibrionales(100)	Desulfovibrionaceae(100)	Bilophila(100)		.759601	1
Otu0124	Firmicutes(100)	Clostridia(100)	Clostridiales(100)	Ruminococcaceae(100)	Hydrogenoanaerobacterium(100)		.641479	1
Otu0125	Firmicutes(100)	Clostridia(100)	Clostridiales(100)	Ruminococcaceae(100)	Pseudoflavonifractor(100)		.015658	1
Otu0126	Firmicutes(100)	Clostridia(100)	Clostridiales(100)	Ruminococcaceae(100)	Flavonifractor(100)		.017547	1
Otu0127	Firmicutes(100)	Clostridia(100)	Clostridiales(100)	Lachnospiraceae(100)	Butyrivibrio(100)		.106234	1
Otu0128	Firmicutes(100)	Clostridia(100)	Clostridiales(100)	Ruminococcaceae(100)	Oscillibacter(100)		.090769	1
Otu0129	Firmicutes(100)	Erysipelotrichia(100)	Erysipelotrichales(100)	Erysipelotrichaceae(100)	Erysipelotrichaceae_incertae_sedis(100)		.304972	1
Otu0130	Firmicutes(100)	Clostridia(100)	Clostridiales(100)	Lachnospiraceae(100)	Syntrophococcus(100)		.351336	1
Otu0131	Firmicutes(100)	Clostridia(100)	Clostridiales(100)	Ruminococcaceae(100)	Flavonifractor(100)		.733134	1
Otu0132	Firmicutes(100)	Clostridia(100)	Clostridiales(100)	Ruminococcaceae(100)	Pseudoflavonifractor(100)		.651459	1
Otu0133	Firmicutes(100)	Clostridia(100)	Clostridiales(100)	Ruminococcaceae(100)	Oscillibacter(100)		.336219	1
Otu0134	Bacteroidetes(100)	Bacteroidia(100)	Bacteroidales(100)	Rikenellaceae(100)	Alistipes(100)		.364453	1
Otu0135	Firmicutes(100)	Clostridia(100)	Clostridiales(100)	Lachnospiraceae(100)	Unclassified(100)		.297057	1
Otu0136	Firmicutes(100)	Clostridia(100)	Clostridiales(100)	Ruminococcaceae(100)	Anaerotruncus(100)		.255234	1
Otu0137	Firmicutes(100)	Clostridia(100)	Clostridiales(100)	Lachnospiraceae(100)	Unclassified(66)		.06594	1
Otu0138	Firmicutes(100)	Clostridia(100)	Clostridiales(100)	Lachnospiraceae(100)	Clostridium_XlVa(100)		.934883	1
Otu0139	Bacteroidetes(100)	Bacteroidia(100)	Bacteroidales(100)	Porphyromonadaceae(100)	Unclassified(100)		.364453	1
Otu0140	Firmicutes(100)	Clostridia(100)	Clostridiales(100)	Lachnospiraceae(100)	Unclassified(100)		.019222	1
Otu0141	Firmicutes(100)	Clostridia(100)	Clostridiales(100)	Lachnospiraceae(100)	Unclassified(100)		.486003	1
Otu0142	Firmicutes(100)	Clostridia(100)	Clostridiales(100)	Lachnospiraceae(100)	Unclassified(100)		.714399	1
Otu0143	Firmicutes(100)	Clostridia(100)	Clostridiales(100)	Lachnospiraceae(100)	Unclassified(100)		.641689	1
Otu0144	Bacteroidetes(100)	Bacteroidia(100)	Bacteroidales(100)	Porphyromonadaceae(69)	Unclassified(69)		.511826	1
Otu0145	Bacteroidetes(100)	Flavobacteria(97)	Flavobacteriales(97)	Unclassified(97)	Unclassified(97)		.718342	1
Otu0146	Bacteroidetes(100)	Bacteroidia(100)	Bacteroidales(100)	Rikenellaceae(100)	Alistipes(100)		.977493	1
Otu0147	Firmicutes(100)	Clostridia(100)	Clostridiales(100)	Ruminococcaceae(100)	Oscillibacter(100)		.345778	1
Otu0148	Firmicutes(100)	Clostridia(100)	Clostridiales(100)	Lachnospiraceae(100)	Unclassified(100)		.08833	1
Otu0149	Firmicutes(100)	Clostridia(100)	Clostridiales(100)	Lachnospiraceae(100)	Clostridium_XlVb(100)		.548781	1
Otu0150	Firmicutes(100)	Clostridia(100)	Clostridiales(100)	Lachnospiraceae(100)	Unclassified(100)		.633538	1

NOTE. Taxonomic classifications for each OTU were generated using the reference taxonomy database from the Ribosomal Database Project. The numbers in parenthesis following each classification show the consistency of the classification assignments (in percent) for all of the sequence reads within a given OTU. p values and q values (false discovery rate adjusted p values, to allow for multiple comparisons) were generated using Metastats software (White JR et al. PLoS Comput Biol. 2009:e1000352).

## Supplementary Materials and Methods

### Microbiota Analysis

DNA was extracted from mouse fecal samples using the FastDNA SPIN Kit for Soil and a FastPrep 24 machine (MP Biomedicals Santa Ana, CA) according to the protocol provided by the manufacturer. Bacterial 16S ribosomal RNA genes were PCR-amplified using barcoded primers MiSeq 27F (5’AATGATACGGCGACCACCGAGATCTACACTATGGTAATTCCAGMGTTYGATYMTGGCTCAG-3’) and MiSeq-338R (5’-CAAGCAGAAGACGGCATACGAGAT-barcode-AGTCAGTCAGAAGCTGCCTCCCGTAGGAGT-3’), which target variable regions V1–V2 of the 16S ribosomal RNA gene. Q5 Taq polymerase (New England Biolabs, Ipswich, MA) was used for the PCR step, and 4 PCR reactions were performed per sample. Cycling conditions were as follows: 98°C for 2 minutes, followed by 20 cycles of 98°C for 30 seconds, 50°C for 30 seconds, 72°C for 90 seconds, and then a final extension step at 72°C for 5 minutes.

The 4 PCR reactions from each DNA extraction then were pooled and concentrated down to 25 μL volumes per sample. PCR amplicons then were quantified using a Qubit 2.0 Fluorometer (Life Technologies, Ltd, Carlsbad, CA) and equimolar concentrations of each were added to a final mastermix for sequencing, using an Illumina MiSeq (San Diego, CA) machine with 2 × 250 bp read length. Sequence data have been deposited in the European Nucleotide Archive under Study Accession Number ERP005850 and Sample Accession numbers ERS459682–ERS459702.

The sequence data were processed by following the MiSeq SOP of the mothur software package (Available at: http://www.mothur.org/wiki/MiSeq_SOP).^1^ Paired-read contigs were created from the forward and reverse read sequence data, and preliminary quality processing was performed by removing all contigs that were shorter than 260 bp, longer than 450 bp, or those that contained any ambiguous bases or homopolymeric stretches longer than 7 bases. Perseus,^2^ as implemented in mothur, was used to remove putative chimeras, and any reads mapping to chloroplasts, mitochondria, eukarya, or archaea also were removed. After these steps, more than 486,000 sequences were left in the final data set (range, 447–51,571 sequences per sample). Next, after a preclustering step (diffs, 3), 97% similarity OTUs were generated. Taxonomic classifications for each of these OTUs were created using the RDP taxonomy provided at the mothur web page. Metastats,^3^ as implemented in mothur, was used to test for significant differences in the proportional abundance of each of the phyla present, and the 150 most abundant OTUs between anti-IL6 and control mice. Bacterial diversity was measured by generating Shannon and inverse Simpson indices in mothur. Before these calculations each sample was subsampled randomly down to 447 reads to ensure equal sequencing depth for each. Kruskall–Wallis and Mann–Whitney U tests, implemented in Minitab v16, were used to test for significant differences in diversity measures between the anti-IL6 and control mouse groups. A cluster dendrogram, using the Bray Curtis calculator, also was generated in mothur from the subsampled data set, and subsequently was visualized using the iTOL online software resource.^4^
